# From RNA design to delivery: Computational strategies for functional RNA therapeutics

**DOI:** 10.1016/j.bioactmat.2026.08.045

**Published:** 2026-08-31

**Authors:** Yiming Wang, Shengxin Tong, Xin Wang, Xiaowen Jin, Duoduo Tan, Yuan Lu

**Affiliations:** a Department of Chemical Engineering, Tsinghua University, Beijing, 100084, China; b State Key Laboratory of Green Biomanufacturing, Department of Chemical Engineering, Tsinghua University, Beijing, 100084, China; c Key Laboratory of Industrial Biocatalysis, Ministry of Education, Department of Chemical Engineering, Tsinghua University, Beijing, 100084, China

**Keywords:** RNA therapeutics, Functional RNA, Delivery systems, Computational design

## Abstract

RNA therapeutics provide a revolutionary means of treating a variety of diseases by precisely regulating gene expression and protein synthesis, with great medical significance. However, there are three key challenges to its clinical application: the inherent instability of RNA, the need for controlled regulation of RNA function, and the lack of an efficient delivery system. Computational strategies provide complementary tools for analyzing and optimizing RNA sequence, structure, function, and delivery, accelerating the rational design of RNAs and the optimization of delivery systems. This review systematically introduces two core advances in the field of RNA therapy: (1) the design and optimization of RNAs based on predictive modeling and algorithmic screening; (2) the intelligent transformation of the delivery system through data-driven methods. Based on these developments, we discuss three future directions for computational RNA molecular design across the dimensions of design algorithms, design mechanisms, and design architectures. This review not only summarizes computational approaches developed to address key challenges in RNA therapeutics, but also highlights opportunities for integrated RNA-delivery co-design. These advances may provide useful insights for the development of next-generation precision therapeutics and their future clinical translation.

## Highlights

• RNA therapeutics offer a transformative approach to treating diverse diseases.
• Instability and inefficient delivery currently hinder clinical translation.
• Computational strategies provide a breakthrough for rational RNA engineering.
• Data-driven advances are reviewed in functional design and delivery optimization.
• A new collaborative framework is proposed to accelerate precision medicine.

## Graphical abstract

**Figure:**
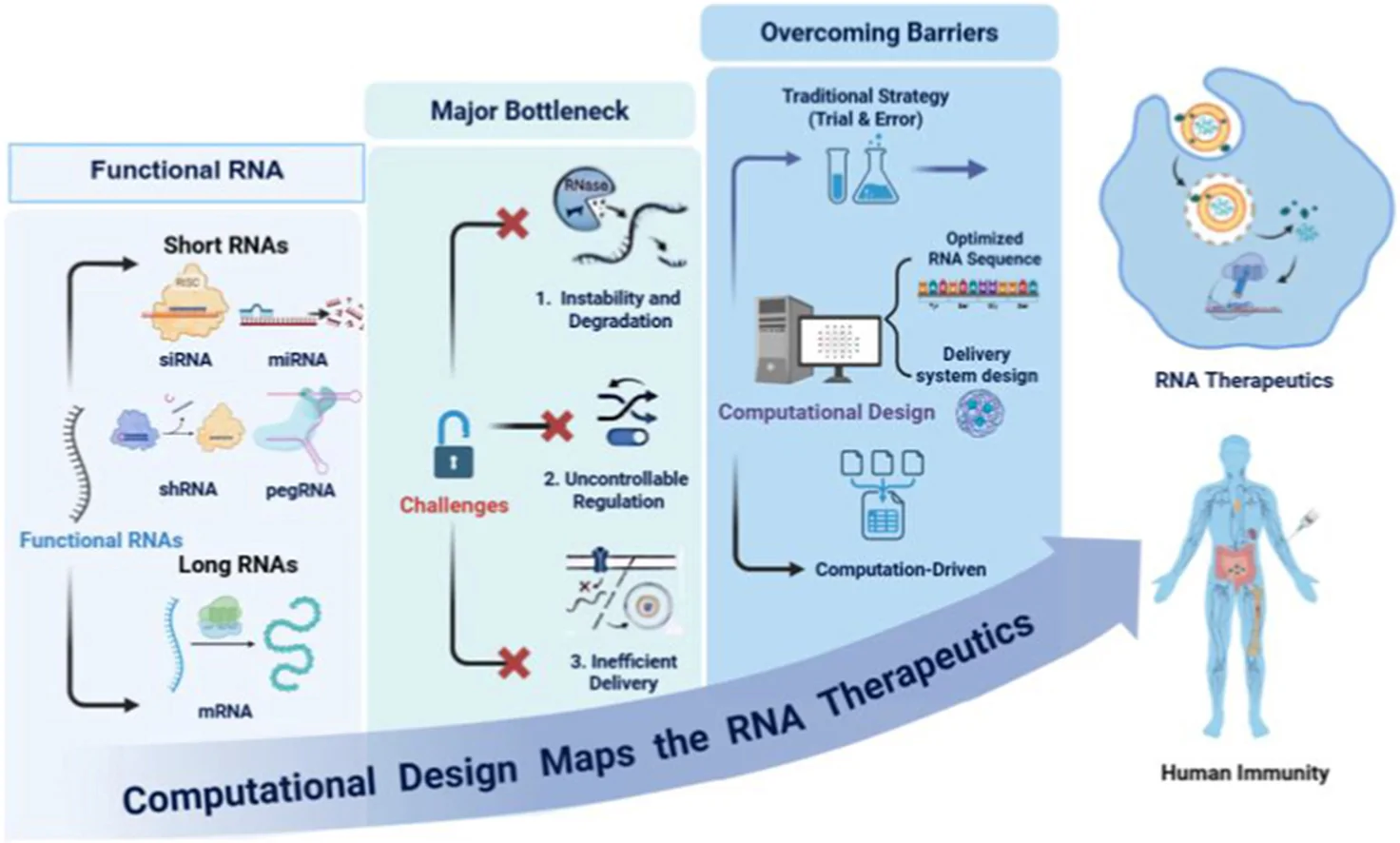

**Table:** Abbreviation Index

Abbreviation	Full name
ACE-ID	Advanced Cellular and Endocytic Profiling for Intracellular Delivery
AIDD	AI-assisted Drug Discovery
ANN	Artificial Neural Network
CADD	Computer-aided Drug Design
CAI	Codon Adaptation Index
circRNA	Circular RNA
CNN	Convolutional Neural Network
CRISPR	Clustered Regularly Interspaced Short Palindromic Repeats
DEN	Deep Exploration Network
DFA	Deterministic Finite-State Automaton
DNA	Deoxyribonucleic Acid
dsRNA	Double-stranded RNA
GalNAc	N-Acetylgalactosamine
geCAI	Gene Expression Codon Adaptation Index
GNN	Graph Neural Network
gRNA	Guide RNA
HTS	High-throughput Synthesis
IRES	Internal Ribosome Entry Site
JCat	Java Codon Adaptation Tool
lightGBM	Light Gradient Boosting Machine
LLM	Large Language Model
LNPs	Lipid Nanoparticles
MINE	Maximal Information-based Nonparametric Exploration
miRNA	MicroRNA
MLP	Multilayer Perceptron
mRNA	Messenger RNA
ODE	Ordinary Differential Equation
ORF	Open Reading Frame
PBPK-PD	Physiologically Based Pharmacokinetic-Pharmacodynamic
PBS	Primer Binding Site
PE	Prime Editing
pegRNA	Prime Editing Guide RNA
RBF	Radial Basis Function
RBP	RNA-binding Protein
RF	Random Forest
RISC	RNA-Induced Silencing Complex
RNA	Ribonucleic Acid
RNAi	RNA Interference
RNase	Ribonuclease
RNN	Recurrent Neural Network
saRNA	Self-amplifying RNA
SHAP	Shapley Additive Explanations
shRNA	Short Hairpin RNA
siRNA	Small Interfering RNA
SVM	Support Vector Machine
TLR	Toll-Like Receptor
UTRs	Untranslated Regions
XGBoost	eXtreme Gradient Boosting

## 1 Introduction

Ribonucleic acid (RNA) serves as a crucial carrier of genetic information in cells and certain viruses, representing an evolutionarily primitive genetic system of deoxyribonucleic acid (DNA) [1,2]. In almost all biological systems, RNA is transcribed from DNA templates and plays multifaceted roles in genetic information transfer, biomass regulation, and protein expression control. Relying on its single-stranded structure, RNA enables localization and interference with the normal functions of other single-stranded nucleic acids through the Watson-Crick base pairing principle, thereby altering the cells' original behavior. As the direct participant in the expression, RNA has a direct impact on the spatiotemporal state of cells. Therefore, RNA has significant potential for disease treatment, and RNA-based therapy is gradually entering the market as an emerging therapeutic paradigm [3].

RNA therapeutics can regulate gene expression or produce therapeutic proteins to target various diseases, such as infectious diseases, cancers, immune diseases, and Mendelian disorders [35]. Its main active ingredients are functional RNAs, which can be divided into short RNAs, long RNAs, and other RNAs. Short RNAs include RNA interference (RNAi), such as small interfering RNAs (siRNAs), short hairpin RNAs (shRNAs), and microRNAs (miRNAs), and Clustered Regularly Interspaced Short Palindromic Repeats (CRISPR)-related guide RNAs (gRNAs). Long RNAs include messenger RNA (mRNA), as well as other RNAs, including self-amplifying RNA (saRNA) and circular RNA (circRNA). The mechanisms of short RNAs primarily involve functional modulation of DNA/RNA, which largely depends on protein-mediated processes. In contrast, long RNAs and other RNAs encode therapeutic elements that are expressed intracellularly through the host cell's translation system. However, regardless of the distinct mechanisms of action, all RNA therapeutics face three major challenges: (1) poor stability due to susceptibility to ribonuclease (RNase) degradation and anionic interference in circulation, (2) realization of controllable regulation of RNA function, and (3) inefficient cellular delivery. Although these three barriers are shared across RNA therapeutics, their relative importance and specific manifestations differ among RNA modalities. For short RNAs, controllable functional regulation is particularly important, involving target selection, silencing or editing efficiency, specificity, and off-target effects, while chemical stability and intracellular delivery remain additional constraints. For mRNA, sequence and structural features are closely associated with both molecular stability and the regulation of protein expression, whereas its relatively large molecular size and physicochemical properties impose additional delivery requirements. For circRNA and saRNA, structural complexity, sustained expression, and modality-specific functional elements introduce additional challenges in molecular design and functional regulation, while their delivery and *in vivo* performance remain important considerations. Delivery-system optimization primarily addresses inefficient cellular delivery, but also influences RNA stability and the effective realization of RNA function after administration. Consequently, the development of effective RNA-based therapy requires both the design of stable RNA constructs and the optimization of delivery systems.

To address the three challenges, traditional strategies typically focus on three main approaches: codon optimization, chemical modification, and delivery system engineering. Codon optimization often relies on empirical rules, utilizing quantitative metrics to assess mRNA stability, albeit with limitations in predictive accuracy [36]. Chemical modifications, including phosphorothioate linkages, base methylation, and 2′-substitutions of the ring, are widely employed to modulate RNA immunogenicity and have been extensively integrated into the *in vitro* production of RNA vaccines [37,38]. Meanwhile, delivery systems such as lipid nanoparticles have been iteratively developed to enhance cellular uptake and facilitate endosomal escape [39]. Although these methods partially mitigate the aforementioned challenges, they remain largely dependent on empirical guidelines and trial-and-error experimentation, resulting in suboptimal efficacy, limited efficiency, and an inability to systematically control RNA sequence and function through principled design. Consequently, as the optimization potential of traditional strategies becomes increasingly constrained, computational approaches are emerging as a promising direction to transcend existing limitations, offering new avenues for the rational design of RNA sequences, structures, and delivery systems.

Computer-aided drug design (CADD) and AI-assisted drug discovery (AIDD) have demonstrated remarkable potential in pharmaceutical development [[40], [41], [42], [43]]. Advances in computational methods have enabled increasingly large molecular and sequence design spaces to be explored efficiently, complementing experimental and rule-based design strategies. RNA therapeutics offer unique advantages for computational design, as they can be conceptually simplified to ordered sequences of four ribonucleotides, making them more suitable for extreme-value control in optimization functions. Furthermore, numerous computational tools for RNA structure prediction and epitope screening have established a robust foundation for computational RNA therapeutic design [[44], [45], [46], [47]]. Although the computational design of delivery systems remains less explored, component optimization of established commercial systems (e.g., lipid nanoparticles) has shown promising progress. These developments highlight how computational designs are creating new opportunities in RNA therapeutics.

**Fig. 1:**
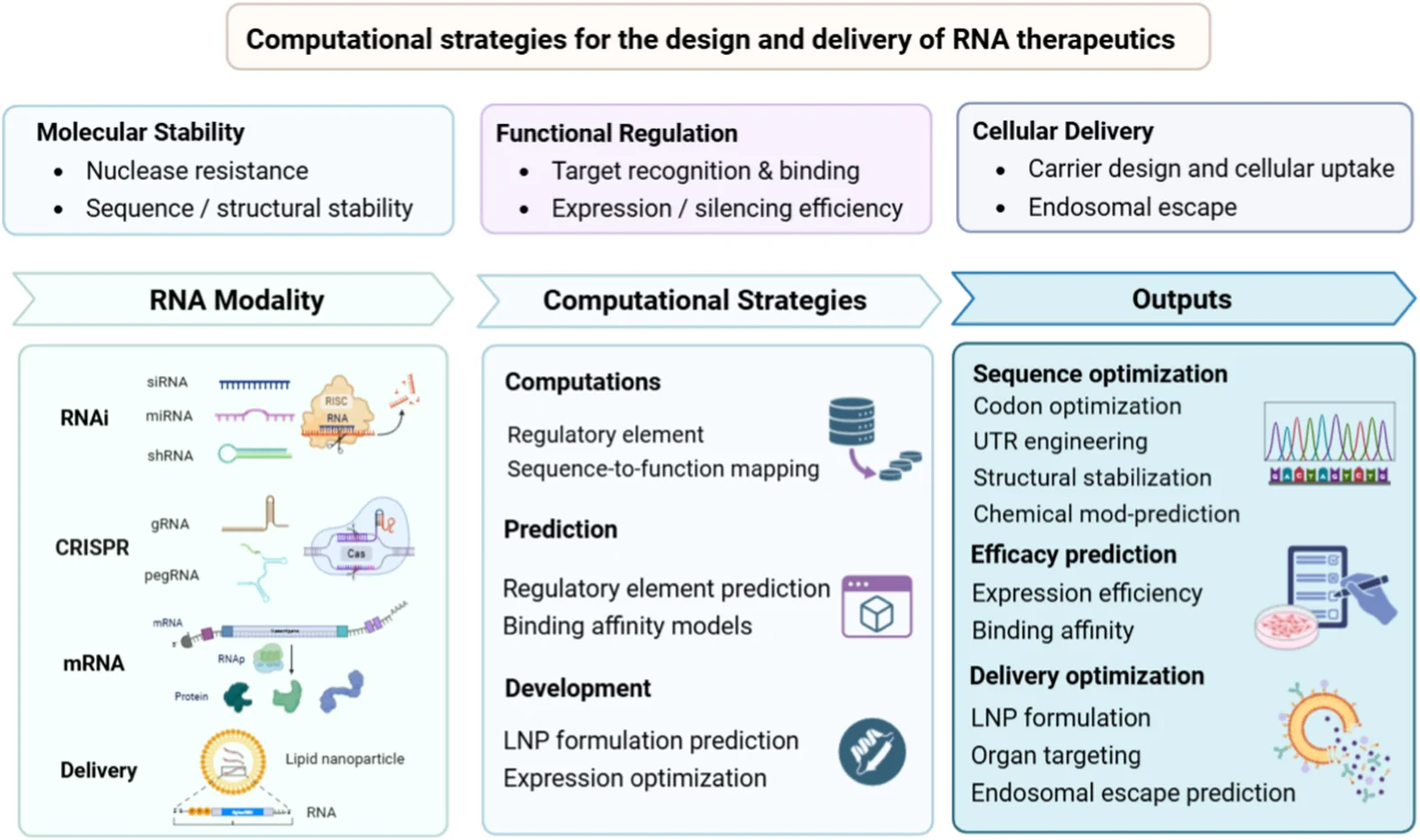
**Organizational map of computational strategies for RNA therapeutic design and delivery.** Functional RNAs are progressively advancing toward clinical application in RNA therapeutics. However, key challenges, such as inherent RNA instability, uncontrolled regulation, and low delivery efficiency, remain critical bottlenecks in the field. Computational strategies empower functional RNAs advancement through computations, prediction, and development. They demonstrate outstanding capabilities in three key aspects: sequence optimization, efficacy prediction, and delivery optimization. Consequently, computational design has significantly accelerated the development of RNA therapeutics. Through the optimization of RNA sequences and the design of delivery system formulations, computational design is driving rapid progress in RNA therapeutics.

Here, the review provides a systematic overview of computational design strategies for optimizing RNA therapeutics and delivery systems (Fig. 1). We systematically categorize design principles for different RNA modalities and summarize relevant design considerations, hereby providing an integrated perspective on RNA design strategies. Furthermore, we highlight an important area in the field by summarizing advances in computational design of delivery systems and exploring their integration with RNA therapeutic development. Finally, we outline future perspectives, highlighting key challenges and emerging directions to guide next-generation RNA therapy innovation.

## 2 Short RNAs design: computational tools, structural prediction, and functional optimization

### 2.1 Computational architecture foundations in functional RNA design

With the rise of data-driven design, computational frameworks have become indispensable tools for functional RNA research. However, various algorithms exhibit differential performance across distinct application scenarios, necessitating a thorough understanding of their core principles prior to implementation. The computational paradigms commonly employed for RNA design can be categorized into two major classes: traditional machine learning and deep learning. Traditional machine learning methods such as random forest (RF) and support vector machine (SVM) are particularly suitable when the available datasets are moderate in size, and the input can be represented by predefined sequence, thermodynamic, physicochemical, or experimentally derived descriptors. Their relatively explicit feature representation can facilitate interpretation of the factors contributing to prediction. However, their performance depends strongly on the quality and completeness of feature engineering, and robustness to overfitting or dataset shift cannot be assumed solely from the choice of algorithm. Their performance can nevertheless be limited when relevant higher-order or spatial relationships are not adequately represented by the predefined input features.

To overcome the bottlenecks in feature extraction, deep learning has enabled the mapping from raw input to functional features through multilayer nonlinear transformations, with distinct architectures emerging in response to the varying data dimensionalities of RNA molecules. Convolutional Neural Networks (CNNs) employ one-dimensional or two-dimensional convolutional kernels with sequence-sliding mechanisms to efficiently extract local nucleotide motifs and spatial adjacency matrix features. These algorithms have demonstrated satisfactory performance in predicting ribosomal loading on 5′ untranslated regions and in characterizing multi-view features of modified small RNAs. However, they exhibit limitations in capturing global interactions when processing long-range sequence dependencies. Graph neural networks (GNNs), including equivariant graph neural networks (EGNNs), abstract nucleic acids or polymer complexes as nodes and edges within three-dimensional space, leveraging message-passing mechanisms to naturally capture the geometric topological constraints of macromolecules. This architecture confers unique spatial advantages in predicting RNA structures and lipid nanoparticle delivery systems. For long-chain RNA molecules, recurrent neural networks (RNNs) and long short-term memory (LSTM) networks can model sequential dependencies, whereas Transformer-based architectures provide an alternative strategy by using self-attention to capture longer-range relationships between sequence positions. Through masked pre-training on large collections of unannotated RNA sequences, pre-trained and foundation models can learn transferable sequence representations that extend beyond local motifs and may support multiple downstream prediction and design tasks. However, their performance remains dependent on the scale and diversity of the pre-training data, the relevance of task-specific fine-tuning data, and downstream validation in the intended biological context. Therefore, these architectures should be regarded as complementary modeling strategies, and model selection should depend on sequence length, data availability, prediction objective, computational resources, and validation requirements.

Model reliability also depends on how predictive performance is evaluated. Validation may range from internal cross-validation or resampling within the development dataset, to held-out testing on unseen samples from the same data source, independent evaluation using separately collected datasets, and direct experimental assessment of computational predictions. These validation settings provide different levels of evidence for model generalizability, particularly when performance is interpreted across studies using different datasets and prediction endpoints.

### 2.2 RNAi-mediated functional RNA

**Fig. 2:**
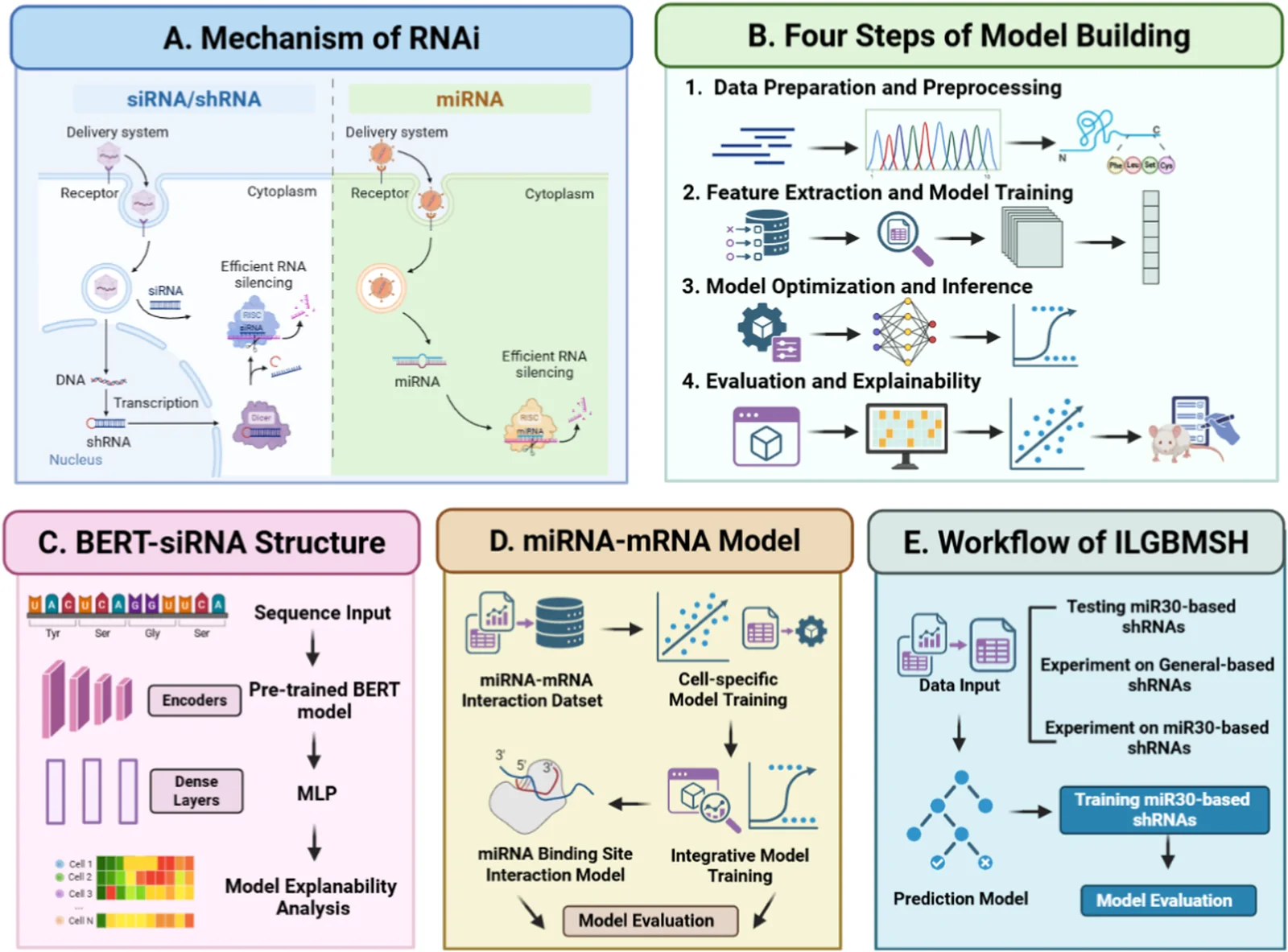
**Mechanisms of RNAi-mediated functional RNAs, the model-building workflows, and algorithm architectures.** (A) Functional therapeutic molecules such as siRNA, shRNA, and miRNA play important roles in RNA therapeutics. Their corresponding mechanisms of action differ and can be selected for suitable scenarios. (B) The construction of models for RNA is generally divided into four steps: data preparation and processing, feature extraction and model training, model optimization and inference, evaluation, and explainability. These four stages constitute the essential phases of RNA model development. (C) Description of the BERT-siRNA structure and model interpretability analysis. The final model output is a sigmoidal value ranging from 0 to 1, representing knockdown efficiency. (D) Study schematic of the miRNA-mRNA interaction model. The model extends a binding-site interaction framework that accounts for interactions between binding sites, thereby promoting more efficient binding to mRNA. (E) The workflow of the ILGBMSH algorithm for the classification model of shRNA target prediction. Panels adapted with permission from: C, ref. [7] Elsevier Limited; D, ref. [21] Elsevier Limited; E, ref. [50] Oxford University Press Limited.

The discovery of RNAi triggered profound and sustained excitement in biomedical research, as it represented the first rapid-response tool capable of modulating cellular phenotypes at the RNA level (Fig. 2A). This mechanism immediately suggested therapeutic potential for gene expression disorders, leading to several clinical-stage candidates [48]. However, many factors also stand in the way, including insufficient specificity, off-target effects, and immunotoxicity [49]. Rational design strategies, such as site-specific modifications, may alleviate these issues but cannot provide a rapid solution, as specificity and off-target effects are profoundly dependent on sequence and structure, which elude accurate prediction by simple empirical rules or experimentation. This bottleneck underscores the critical reliance on computational methods for designing target RNA sequences. Currently, emerging computational frameworks for RNAi-mediated RNA can rapidly identify optimal sequences through non-rational paradigms, focusing primarily on: (i) the *de novo* design or optimization of RNA sequences and structures, (ii) target prediction and disease association, and (iii) the minimization of off-target effects through genome-wide specificity analysis. For siRNA, the primary focus is the prediction and optimization of silencing efficacy, sequence specificity, and chemical-modification effects. For miRNA, the major computational tasks shift toward target identification, regulatory-network analysis, and disease-association prediction because of its endogenous and one-to-many regulatory behavior. For shRNA, computational design additionally involves the relationship between precursor sequence and structure, processed siRNA activity, and backbone-dependent performance. This part systematically explores these key computational frontiers, highlighting the application of computational methods in designing RNA sequences and providing a critical evaluation of existing methodologies (Fig. 2B). These modality-specific objectives are further reflected in the input data, computational methods, validation evidence, and major limitations discussed in the following subsections.

#### 2.2.1 siRNA

siRNA typically consists of 19 base pairs and two nucleotide overhangs at the 3’ end. Efficient silencing relies on accurate, rapid, and predictable sequence design. However, siRNA design is primarily constrained by three factors. (1) Previous sequence optimization has depended on empirical rules derived from extensive experimental data, whose accuracy and applicability are not guaranteed. (2) With the substantial increase in candidate sequence data, scoring mechanisms based on qualitative indicators have gradually become insufficient, necessitating stricter screening criteria to approach the optimal sequence. (3) Necessary chemical modifications to improve pharmacokinetics and immunogenicity can affect silencing efficiency, underscoring the need to account for the deviations they introduce. Accordingly, the central computational objective for siRNA is to prioritize candidate sequences with favorable silencing activity and target specificity while accounting for sequence-, structure-, and chemical-modification-dependent effects. Computational methods, by extracting meaningful information from large-scale data, integrating multi-dimensional data for efficient and precise prediction, and screening optimal sequences from massive datasets, have become a core tool for systematically addressing these challenges (seeTable 1).

**Table 1:** **Computational methods used for siRNA design and prediction.** Specific topics, models, algorithms, datasets, input features, prediction endpoints, reported performance, validation evidence, code/web tool availability, and studies are organized.

Topic	Models	Algorithm	Datasets	Input Features	Prediction Endpoints	Reported Performance	Validation Evidence	Code/Web Tool Availability	Critical Assessment	Studies
siRNA design	---	Multi-layer feed forward networks	Training dataset: a heterogeneous set of 653 siRNAs collected from the literature Validation dataset: siRNA dataset recently published by Novartis	3 or 4 optimized physicochemical parameters	siRNA-mediated gene silencing efficiency	Correlation coefficient R = 0.75 (Neural Network) and R = 0.744 (Linear Regression) on the Novartis validation set	Internal: non-overlapping cross-validation; External: independent Novartis dataset containing 2431 siRNAs	NCBI ftp site	Lack of chemically modified siRNA prediction	[4]
siRNA design	---	Radial Basis Function (RBF) network and decision tree learning and their combined method	Training dataset: a total of 833 known effective siRNA sequences (effective siRNA sequences); the 847 worst-ranked ineffective siRNAs from Huesken et al. [5] (Ineffective siRNA sequences) Testing dataset: the recently reported effective and ineffective siRNAs	Point-specific positional nucleotides	Estimated gene-silencing efficacy probability for candidate siRNA sequences	Predicted identification rates (82.4% for effective and 23.8% for ineffective siRNAs) of the combined method (RBF + DT) on the independent testing set	External testing on separately reported effective and ineffective siRNA datasets for multiple genes	From the author	Absence of explicit mRNA structural context	[6]
siRNA target prediction	BERT-siRNA	A BERT module	The largest siRNA dataset from Huesken [5]	Raw 19-nt siRNA sequence features without artificial preprocessing	A continuous Sigmoid layer output score ranging from 0 to 1	Spearman's correlation = 0.639, Pearson's = 0.611; AUPRC = 0.714, AUROC = 0.826	External: independent public siRNA dataset; experimental corroboration with reported PDCD1, CD38, and IL6 knockdown results.	GitHub	Uncertain transferability to other sequence formats	[7]
Chemically modified siRNA activity prediction	---	A random forest (RF), a multilayer perceptron (MLP), artificial neural network (ANN), and a linear support vector machine (SVM)	The training and testing datasets were derived from the siRNA-mod database	One-hot encoded continuous arrays	Binary classification (efficacious vs. inefficacious)	On the external validation dataset at the 70% biological inhibition threshold (ROC AUC = 0.895, AUPRC = 0.921)	Internal: 5-fold cross-validation; External: independent external validation dataset	No	Limited representation of rare chemical modifications	[8]
Chemically modified siRNA activity prediction	Cm-siRPred	A cross-attention model and a two-layer convolutional neural network (CNN) module	siRNAs from the siRNA mod-database	Three heterogeneous views: One-hot encoded sequences, 166-dimensional MACCS molecular fingerprints of 128 modifications, and a 10-dimensional continuous physicochemical property matrix	Continuous predicted cm-siRNA silencing efficiency score outputted via a Sigmoid node	In 5-fold cross-validation metrics (PCC = 0.8283, SCC = 0.8263, AUC = 0.9147, MAE = 0.1152, MSE = 0.0288)	Internal: cross-validation; External: independent dataset; case-study corroboration using approved siRNA drugs	A freely accessible interactive web server	Limited representation of rare or unseen modification patterns	[9]

To address the applicability limitations of empirical rules, the advancement of rational design has been driven by the systematic summarization of empirical rules derived from extensive experimental data, particularly in sequence-based target optimization. By analyzing large sets of effective siRNA sequences, nucleotide preferences at each position have been identified and subsequently validated. Building on this foundation, these design rules have been integrated into comprehensive scoring systems, enabling systematic discrimination and marking the beginning of computational sequence design [51]. The criteria used to designate siRNA sequences as “effective” are primarily based on quantitative knockdown efficiency thresholds, typically requiring a normalized reduction of 70% or 80% in target mRNA expression. However, these screening processes frequently generate substantial false positives. To systematically refine these systems, researchers have progressively incorporated highly specific sequence and structural parameters. These include thermodynamic asymmetry indicators, such as the ΔG difference between positions 1 and 18, specific nucleotide composition constraints, such as AU-rich 5′ termini in the antisense strand, and avoided dinucleotide content indices to minimize cytotoxicity. Subsequent improvements in design flexibility were achieved by weighing various influential factors [52]. Through the incorporation of additional evaluation parameters, the integration of thermodynamic and sequence-based approaches improved the concordance between predicted and experimental results to 75%, while enabling optimal target analysis across 22,600 human mRNAs [4]. With the continuous expansion of screening rules, filters for innate immunotoxicity and cytotoxicity were progressively refined [53,54]. The subsequent proliferation of online design software extended these capabilities to genome-wide design applications [55]. These rule- and scoring-based approaches remain useful for rapid candidate filtering when experimentally derived design principles are available and model interpretability is important. However, their performance is constrained by predefined features and weighting schemes, and candidates with similar scores may still exhibit substantially different silencing activities. Therefore, such methods are more appropriate for initial sequence prioritization than for resolving complex nonlinear sequence–activity relationships, motivating the subsequent use of data-driven prediction models.

An essential component of sequence design involves evaluation modules, where machine learning approaches have significantly advanced assessment methodologies. High-performance screening modules enable precise identification of optimal sequences from extensive candidate pools. Conventional guideline-based scoring strategies often prove inadequate for rigorous screening, frequently yielding numerous seemingly viable but suboptimal design alternatives [6]. In contrast, machine learning algorithms progressively converge toward optimal solutions through iterative refinement of previous results. Bayesian approaches estimate conditional relationships between sequence motifs and silencing outcomes and are relatively interpretable when predefined sequence features are available [56]. Their usefulness, however, depends on whether the assumed probabilistic relationships adequately represent the application dataset. More flexible nonlinear relationships can be modeled using machine-learning approaches. For example, Radial Basis Function (RBF) networks combined with decision-tree learning use position-specific nucleotide information to predict siRNA activity and have been evaluated using separately reported siRNA datasets that were not used for model development [6]. Sequence-based deep-learning models reduce reliance on manually selected descriptors by learning representations directly from raw siRNA sequences. By visualizing attention scores and hidden layers, they were further evaluated on an independent public siRNA dataset, providing external evidence for their predictive performance (Fig. 2C) [7]. This approach is particularly suitable when sufficiently large sequence–activity datasets are available, and the objective is to learn complex sequence patterns without extensive manual feature engineering. However, the current implementation was developed and evaluated primarily for 19-nt siRNA sequences, and its generalization to other sequence formats remains to be established. Accordingly, feature-based machine-learning and sequence-based deep-learning approaches should be regarded as complementary choices.

siRNA possesses the capacity to induce innate immune responses, necessitating modulation through chemical modifications [57]. Upon administration, exogenous siRNA enters the body and, through the mediation of Toll-like receptors (TLRs) or cytoplasmic RNA sensors, triggers the activation of type I interferons and pro-inflammatory cytokines [58]. To alleviate this issue, 2′-O-methyl (2′-OMe) and 2′-fluoro (2′-F) substitutions at the ribose 2′ position have been widely adopted. These modifications alter the spatial conformation and binding steric hindrance of siRNA, thereby disrupting hydrogen bonding and structural recognition between siRNA and immune receptors. Consequently, they effectively prevent TLR activation by siRNA, enabling immune evasion. However, the introduction of these modifications perturbs the thermodynamic structure of the original sequence. Most existing efficacy prediction models fail to account for these structural perturbations, thereby limiting the applicability of models that do not explicitly represent chemical modification patterns. To address this problem, Dominic Martinelli pioneered the application of three machine learning approaches (random forest, multi-layer perceptron artificial neural networks, and support vector machine) to predict the silencing activity of chemically modified siRNAs based solely on sequence information and modification patterns [8]. Subsequently, a multi-view learning strategy was implemented to predict the efficacy of chemically modified siRNAs, with the algorithm demonstrating favorable results in cross-dataset validation and case studies of approved siRNA therapeutics [9]. However, this strategy exhibits strong data dependency, as the classification accuracy and generalization boundaries of the model are constrained by the scale and quality of modified samples within the dataset. Notably, sequence design itself can also modulate siRNA immunogenicity, minimizing immune receptor recognition while preserving silencing activity. This facilitates the auxiliary screening of siRNA sequences and the development of RNA vaccine adjuvants, thereby establishing a foundation for the rapid development of nucleic acid vaccine adjuvants [59].

siRNA computational design has progressed from empirical and thermodynamic scoring rules to statistical, machine-learning, and deep-learning approaches. The principal inputs include sequence composition, physicochemical and thermodynamic features, structural information, and, in more recent models, chemical-modification patterns. Validation ranges from internal cross-validation to independent datasets and selected experimental or therapeutic case studies. Nevertheless, model performance remains dependent on dataset composition and experimental context, particularly for chemically modified siRNAs.

#### 2.2.2 miRNA

miRNA typically consists of 19-24 nucleotides and functions by binding to the 3′UTR of mRNA, leading to its degradation or translational inhibition [60]. The mechanism of action of miRNA is similar to that of siRNA, yet they differ in target recognition and biogenesis pathways. As essential endogenous regulatory molecules, aberrant miRNA expression is closely associated with numerous diseases. Consequently, the inherent “one-to-many” regulatory characteristic of miRNA poses significant challenges for sequence design. Therefore, unlike siRNA, the major computational objective for miRNA is not limited to direct sequence optimization, but extends to the identification of biologically relevant targets, reconstruction of regulatory relationships, and prediction of disease associations. This raises several critical issues. First, confidently identifying mRNA targets directly regulated by miRNA on a genome-wide scale remains a major challenge. Additionally, linking complex target gene networks to specific diseases and establishing causal relationships between miRNA expression levels and pathological conditions presents further difficulties. Traditional experimental validation methods, characterized by low throughput and high cost, are often inadequate for addressing these systemic challenges. Computational approaches address these tasks using different types of biological information, ranging from sequence complementarity and conservation to experimentally annotated interactions, regulatory networks, and multi-omics data (seeTable 2). Consequently, the appropriate modeling strategy depends strongly on whether the objective is direct target prediction, network-level relationship inference, or disease-association analysis.

**Table 2:** **Computational methods used for miRNA design and prediction.** Specific topics, models, algorithms, datasets, input features, prediction endpoints, reported performance, validation evidence, code/web tool availability, and studies are organized.

Topic	Models	Algorithm	Datasets	Input Features	Prediction Endpoints	Reported Performance	Validation Evidence	Code/Web Tool Availability	Critical Assessment	Studies
Human miRNA prediction	ProMiR	A probabilistic co-learning method based on the paired hidden Markov model	Positive dataset: 5-fold cross-validation with a positive dataset [known human miRNA precursor (pre-miRNAs)] Negative dataset: 1000 extended stem–loop structures randomly extracted from human chromosomes	Two-dimensional joint probability feature matrix based on nucleotide sequences and Watson-Crick secondary base-pairing states	Binary classification probability score for functional pre-miRNA candidates and exact cleavage boundary predictions for mature miRNA regions	Sensitivity = 73%; specificity = 96% in 5-fold cross-validation	Internal: 5-fold cross-validation; Experimental: qPCR and Drosha-RNAi validation confirmed 9 of 23 predicted candidates in HeLa cells	Web server platform	Diminished prediction sensitivity for atypical variants characterized	[10]
miRNA identification	BayesmiRNAfind	Naïve Bayes classifier	Positive examples: known miRNAs Negative examples: highly conserved regions, as defined by homology using the BLAT sequence alignment	Hybrid high-dimensional vector combining 62 secondary structure features and 12 sequence-based motif words	Posterior probability score indicating the likelihood of a 21-nt candidate window belonging to the mature miRNA class	Peak cross-validated classification accuracy (Area Under the ROC Curve, AUC = 0.978 to 0.986)	Internal: repeated train/test resampling and 5-fold cross-validation	Web server platform	Dependency on the strict conditional independence assumption of Naïve Bayes metrics	[11]
miRNA Target Prediction	MiRTarVis	Bayesian inference, MINE analyses, and conventional correlation and mutual information analyses	MicroRNA-mRNA expression profile data	Heterogeneous multidimensional data	Continuous multi-algorithm index scores quantifying the intensity of negative regulatory associations	---	Implementation verification against the original GenMiR++ Matlab output; case-study application to expression-profile datasets	Web server platform	Pre-existing static sequence databases restricts analysis of novel variant single nucleotides	[12]
miRNA binding sites	MiRWalk	TarPmiR algorithm [13]	All mRNA sequences and other necessary information of all known genes of human, mouse, rat, and cow	Multi-parametric sequence-structure matrices encompassing complete transcripts	Binding Probability Score	---	Database/resource application; no independent external validation of miRWalk reported	Web server platform	Lacking of self-supervised language-model abstraction capabilities to detect unannotated non-linear structural variations	[14]
miRNA Target Prediction	---	Support vector machine classifiers	Matched miRNA and mRNA RNA-seq data (level 3.0) from TCGA for TGCT and KIRC	Multi-view feature vectors combining target site binding parameters and gene regulatory network	Discrete binary classification status for unknown candidate miRNA-mRNA pairs	Reported 30–50% improvement in precision over TaLasso and GenMiR++ across the TGCT and KIRC datasets	Internal: 10-fold cross-validation on the training data; Held-out: evaluation on top-ranked TaLasso/GenMiR++ interaction sets separated from the training data within the same TCGA datasets	Online	Severe feature redundancy	[15]
Human miRNA targets prediction	DeepMirTar	Stacked denoising auto-encoders	Positive dataset: mirMark data [16] and CLASH data [1] Negative data: generated using mock miRNAs similar to a previously described method [16]	A comprehensive 750-dimensional feature vector hierarchical matrix	miRNA targeting interactions probability score	Test auROC = 0.9793, overall accuracy ACC = 0.9348, TPR = 0.9235, TNR = 0.9479	Held-out: test subset from the development dataset; External: independent PAR-CLIP-derived 48-site dataset	GitHub	Lacking of direct mechanistic visualization or precise biophysical tracking	[17]
Human miRNA Target Sites identification	---	Deep learning methods	Processed CLASH data [1]	Raw sequence input descriptors	A classification layer ranging from 0 to 1	Test auROC = 0.9440	Held-out test evaluation on the information-balanced ground-truth dataset	Online	Limited to specific species	[18]
Human lncRNA-miRNA interactions prediction	GCNCRF	Graph convolutional neural network and conditional random field	LncRNA-miRNA interaction pair collected	Heterogeneous network matrices: Integration embedding an adjacency matrix	A connection score reconstructing the bipartite lncRNA-miRNA interaction	5-fold cross-validation average ROC AUC = 0.9470, recall = 0.8795, specificity = 0.9254	Internal: 5-fold cross-validation.	GitHub	Different from the actual situation, showing false positives	[19]
miRNA-mRNA interaction efficiency prediction	MiBSIM	Elastic net regularization	Transfection data from HeLa and HCT116 cells [13], HeLa and HEK293FT cells [20]	A comprehensive biophysical feature matrix	Measured log-fold change	A statistically significant 6.43% absolute boosting in Pearson correlation over traditional baseline models	Internal: 100 bootstrap resamplings using 80% training/20% test splits; External: evaluation on HEK293FT and HCT116 datasets	Online	Different from the environment inside the body	[21]

Currently, numerous computational methods have been applied to miRNA target prediction. To date, few experimental techniques can reliably confirm miRNA regulation, making computational approaches a primary means for predicting miRNA-mRNA interactions with significant utility [61]. Early computational methods, such as TargetScan, were based on sequence complementarity and the identification of conserved sequences [62]. By aligning miRNA sequences to complementary regions in the 3’ UTRs of mRNAs and conducting cross-species genomic comparisons, conserved binding sites were identified. Although this approach substantially narrowed the scope for experimental validation, it still suffered from a high rate of false positives.

With the continuous advancement of computational methods, predictive models have gradually evolved from purely sequence-based algorithms to multidimensional, machine-learning-driven models. Early pioneering work by Nam et al., in 2005 employed a probabilistic co-learning model to predict miRNAs in the human genome, achieving 73% sensitivity and 96% specificity [10]. Yousef et al. subsequently applied Bayesian classification algorithms to analyze miRNA genes across multiple species, obtaining 83% sensitivity and 96% specificity in predictions for the *C. elegans* genome [11]. Such probabilistic approaches can integrate sequence evolutionary features and secondary structural stability and are therefore useful when these predefined biological features are informative. However, their dependence on model assumptions, including canonical sequence and structural patterns, can limit their applicability to atypical or non-canonical cases. These advances in miRNA gene identification further stimulated demand for improved target prediction methods. The development of miRTarVis represented a significant advancement by incorporating GenMiR++ and MINE (Maximal Information-based Nonparametric Exploration) analyses to enable visualization and prediction of miRNA targets within expression profiles [12]. The results exhibit strong data dependency. Traditional machine learning algorithms and deep learning algorithms, including random forests [14], unsupervised learning [15], deep learning [17,18], and convolutional neural networks [19], have subsequently been applied to miRNA target prediction. Compared with rule- or probabilistic-feature-based approaches, these data-driven models can capture more complex relationships when sufficiently representative annotated datasets are available, but their reliability remains sensitive to training-data quality, feature representation, and the biological context represented in the dataset [21].

miRNAs, as endogenously expressed small non-coding RNAs, have been shown to be associated with various specific diseases [63]. The detection of circulating miRNAs in blood has emerged as a mainstream paradigm for miRNA-driven therapeutic strategies. The prediction of miRNA–disease associations is commonly formulated as a classification or association-inference problem, for which machine-learning methods have been widely applied. However, computational reliability is strongly affected by noise, incompleteness, and sparsity in available association datasets. To address this challenge, Yu et al. pioneered the application of the GLNMDA method, which reconstructs miRNA similarity matrices and disease similarity matrices to infer potential associations [64]. Such similarity-based methods are useful when sufficiently informative known associations and similarity relationships are available, but their predictions remain dependent on the completeness of these prior data. Furthermore, the inductive matrix completion model has shown promising results in predicting miRNA-disease associations for specific cancers, including colon cancer, kidney cancer, and lung cancer [65]. However, such models are unable to extrapolate predictions for diseases or molecules without any known association information, necessitating further methodological optimization.

For miRNA-disease association prediction, graph-based models provide a suitable representation when the available data contain explicit relationships among miRNAs, diseases, genes, or other biological entities [66]. Li et al. pioneered the application of graph convolutional networks by developing HGCNMDA, which circumvented the complexity of similarity-based methods and eliminated the cumbersome reliance on manually curated similarity matrices. The model achieved an AUC of 0.9626 [67]. Subsequent advancements in graph neural networks and hypergraph neural networks have led to significant progress in deep mining of miRNA-disease associations. The PDMDA framework was the first to employ graph neural networks for predictions based on miRNA sequence and structural features [68]. MVNMDA established a general framework for disease prediction by constructing multiple views, thereby overcoming limitations associated with data dependency [69,70]. These approaches are particularly useful when sufficiently rich and complementary data sources are available, but their increased representational capacity also results in greater dependence on high-quality, large-scale, and consistently annotated datasets. Sparse associations, incomplete data views, and previously unseen miRNAs or diseases can therefore reduce generalizability, while model complexity also increases computational requirements. Further approaches have focused on improving feature extraction and information-fusion strategies [71,72]. Additionally, interactions between miRNA-mRNA (Fig. 2D) and miRNA-miRNA have revealed the therapeutic potential of miRNAs [21,73].

Overall, miRNA modeling relies on more heterogeneous biological information than siRNA efficacy prediction, including sequence complementarity, expression profiles, interaction networks, similarity information, and multi-omics data. Correspondingly, computational approaches have expanded from rule-based and probabilistic methods to machine learning, graph-based learning, and multi-view data integration. Validation includes cross-validation, independent datasets, and selected experimental evidence, although the level of validation varies substantially among studies. Major limitations arise from incomplete interaction annotations, heterogeneous datasets, limited causal information, and reduced generalizability to previously uncharacterized miRNAs or diseases. Thus, in miRNA research, the principal computational challenge lies primarily in robust target and disease-context prediction rather than in sequence optimization alone.

#### 2.2.3 shRNA

Short hairpin RNA (shRNA) typically serves as the primary transcript of siRNA, enhancing its stability and heritable silencing effects, making it a key tool for achieving long-term, stable gene silencing. However, the efficient and rapid design of shRNA is hindered by several bottlenecks. (1) It is difficult to accurately predict the final silencing efficiency of the siRNA processed from the primary shRNA sequence. (2) There is a lack of robust design methods that can simultaneously address high-throughput genome-wide applications and personalized design tailored to specific backbone structures. (3) Rapid large-scale screening and interpretable classification of shRNA remain challenging. Accordingly, shRNA represents a multilevel computational design problem in which precursor sequence and structure, backbone context, and the silencing activity of the processed siRNA need to be considered together. Computational design offers a powerful paradigm for systematically addressing these issues through model prediction and the integration of large-scale algorithmic approaches, driving the evolution of shRNA design from a trial-and-error mode based on limited experience toward a data- and algorithm-driven paradigm.

In the early stages of shRNA design, reliable predictive tools were lacking. Although more mature guidelines for the rational design of shRNA were later developed, activity assessment remained heavily dependent on experimental validation [74]. To address this issue, Beisel et al. pioneered the development of a computational evaluation system for shRNA efficacy through mathematical modeling [75]. Although this system showed some discrepancies with actual shRNA folding patterns, such mechanistic modeling remains useful when precursor folding and processing constraints need to be explicitly considered, but its reliability is limited when the assumed structures deviate from experimentally observed conformations. Interestingly, subsequent studies demonstrated that recognition of sequence features characteristic of functional siRNAs enabled rational shRNA design with 70% efficacy [76], revealing a measurable correlation between siRNA and shRNA sequences that informs design principles. Sequence-based rules can therefore provide a practical starting point for shRNA design when processed-siRNA features are informative, although they do not fully account for precursor- and backbone-dependent effects.

With the expansion of requirements to genome-wide scales and specific application scenarios, achieving high-throughput, customized, optimal design has become a new challenge. On one hand, the development of Massively Parallel Sensor Assay has extended the functional identification of shRNA and the screening of efficient sequences to the genomic level, generating massive shRNA datasets that lay the foundation for the large-scale data requirements of machine learning [77]. On the other hand, algorithmic models have begun to be optimized for specific needs. For instance, by analyzing miR30-based shRNA sequences, unique sequence preferences have been identified. Building on this, the development of the miR_Scan model has established a foundation for designing highly efficient and specific shRNA targeting sequences [78].

To further enhance prediction accuracy, generalization capability, and model interpretability, machine learning has been applied to shRNA sequence design and target prediction. SplashRNA enables the design of effective shRNA based on miRNA templates [79]. This approach utilizes sequential learning algorithms to learn from both unbiased and biased datasets, demonstrating >85% protein knockdown for selected designs in the reported evaluation. However, the model is applicable to the batch high-throughput design of microRNA-backbone-based shRNA libraries, yet it exhibits significant backbone-specific data dependency, resulting in predictive blindness when applied to general vector backbones due to environmental shifts. To overcome the limitations of single machine learning models in feature fitting and to incorporate nonlinear macromolecular microenvironmental features, Zhao et al. developed an interpretable classification system incorporating multiple biological features of shRNA sequences through deep learning, achieving an exceptional Pearson correlation coefficient of 0.985 by ILGBMSH algorithm (Fig. 2E) [50]. It currently provides high-performing prediction and design capabilities for shRNA therapeutics. This approach still faces convergence uncertainties arising from the lack of high-quality data.

Machine learning algorithms have provided tools for the efficient prediction and precise design of shRNA, yet they remain subject to certain limitations. First, the majority of models are heavily dependent on biased datasets, characterized by a paucity of negative data, over-reliance on chemical synthesis, and confinement to specific shRNA backbones, which results in insufficient generalizability and suboptimal cross-system predictive accuracy. Second, the highly dynamic nature of the *in vivo* environment, compounded by interference from diverse molecular species, introduces discrepancies between computational designs and actual folding conformations. Thermodynamic and sequence-based algorithms predicated on idealized static states often fail to faithfully reflect biological efficacy *in vivo*. Finally, sequence preferences arising from random library construction, cytotoxicity induced by distinct promoters, and the physical constraints imposed by shRNA switches in terms of length and induction range represent additional challenges that warrant future investigation.

These three RNAi-related modalities illustrate how different biological functions and structural contexts lead to distinct computational requirements. Although the specific input features and modeling strategies vary, their predictive performance is consistently constrained by dataset quality, experimental context, and validation depth. In addition, most current approaches focus primarily on sequence- or molecule-level optimization, while delivery-related variables are generally treated separately. This separation remains an important limitation for translating computationally optimized RNA designs into therapeutic applications.

### 2.3 RNA-programmable CRISPR systems

Since its introduction in 2012, CRISPR gene editing technology has emerged as one of the most groundbreaking scientific breakthroughs in the life sciences. Precise and reliable gene editing holds fundamental therapeutic potential for genetic disorders. As the critical targeting component of CRISPR systems, the design and optimization of gRNA sequences are pivotal for minimizing off-target effects while maximizing editing efficiency. In contrast to RNAi-related molecules, which regulate gene expression primarily through RNA-mediated silencing, CRISPR-associated guide RNAs function as programmable targeting components whose sequence design directly influences editing efficiency and specificity. Accordingly, computational design in this context is centered on identifying guide sequences that balance on-target activity with off-target risk, while the relevant design variables and modeling complexity depend on the specific editing system. Currently, computational approaches for gRNA design primarily follow two paradigms: (1) scoring-based prediction of targeting efficiency using design rules derived from traditional rational design principles, and (2) machine learning and deep learning methods that extract predictive features from effective sequences to design high-efficiency guides. These approaches have been implemented in numerous web-based platforms, whose development and applications have been comprehensively reviewed [80,81].

**Fig. 3:**
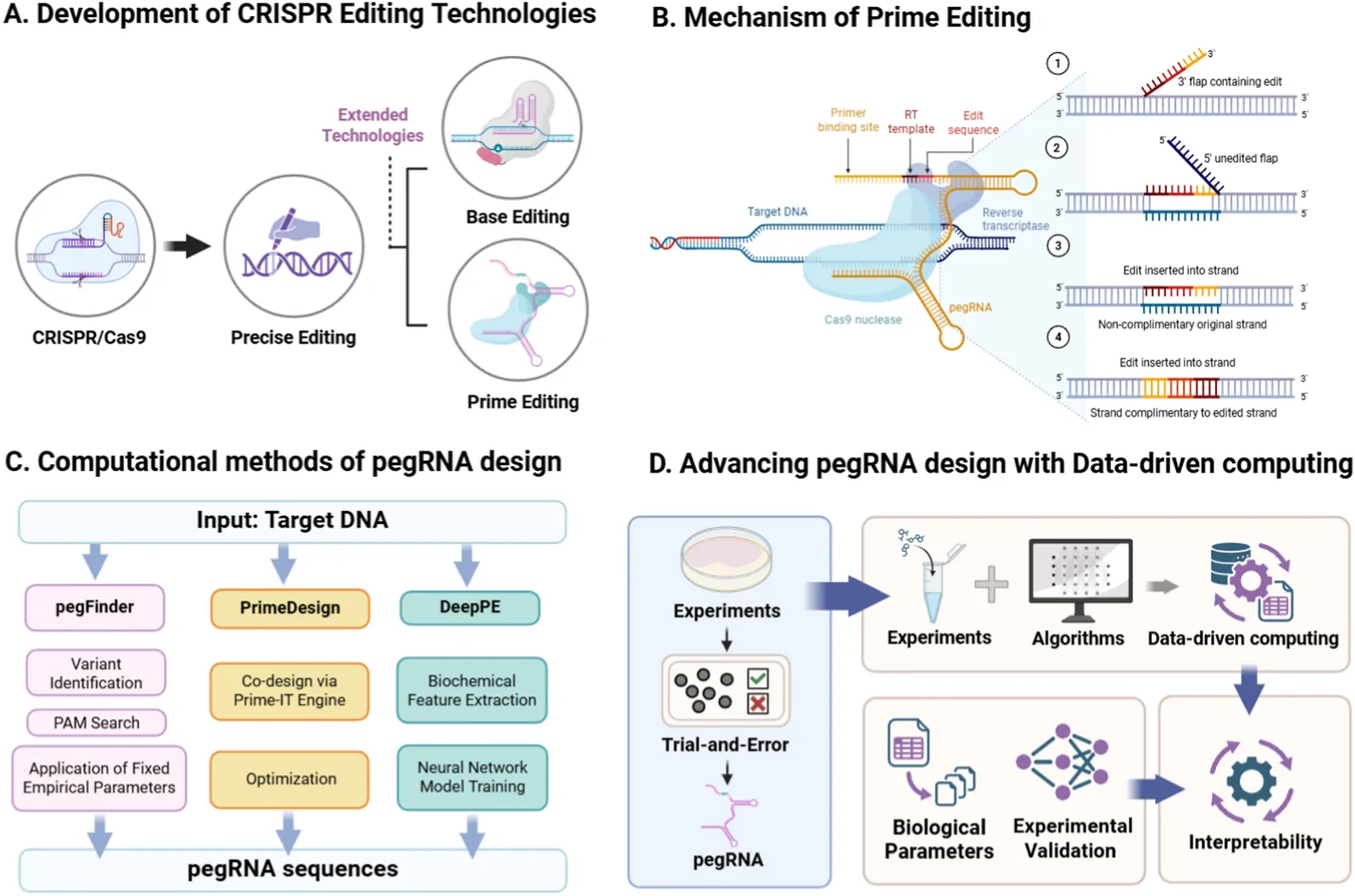
**Evolution and mechanisms of prime editing, the computational designs and optimization of pegRNA.** (A) The evolution of CRISPR technology toward precision editing. With continuous optimizations, CRISPR has branched into base editing and prime editing. Prime editing currently serves as the mainstream approach due to its broader applicability. (B) The prime editing process involving prime editor and pegRNA of target DNA. (C) Workflows of the pegFinder, PrimeDesign, and DeepPE models. All three models utilize a target DNA sequence as input to generate the desired pegRNA sequence as output. (D) Data-driven computational design of pegRNA. Given the limited pegRNA data currently available, most developments rely on a trial-and-error approach. Therefore, it is essential to integrate experimental data with computational algorithms. By optimizing biological parameters and incorporating experimental validation, the design workflow is continuously refined to achieve rapid generation and model interpretability. Panels adapted with permission from: B, ref. [83] under a Creative Commons license CC BY 4.0; C, ref. [84,85] Springer Nature Limited; ref. [86] under a Creative Commons license CC BY 4.0.

Extended editing technologies based on CRISPR are continuously evolving (Fig. 3A). Prime editing (PE editing), which employs an engineered SpCas9 and a reverse transcriptase (Moloney murine leukemia virus reverse transcriptase, M-MLV RT), enables versatile and highly precise editing in mammalian cells and has emerged as a promising technology in the field of gene editing therapy (Fig. 3B) [82,83]. PE editing imposes almost no sequence constraints on the editing target and can directly copy new genetic information into the designated genomic locus. In this process, the prime editing guide RNA (pegRNA) serves as a critical RNA component, functioning as both a primer binding site (PBS) and a template for reverse transcription, with an additional 3′ extension that hybridizes to the nicked target DNA. The efficiency of prime editing depends heavily on pegRNA design, and, as with gRNAs, the pegRNA sequence significantly influences editing outcomes. Compared with conventional gRNA design, pegRNA optimization therefore represents a higher-dimensional design problem because multiple sequence elements and their interactions must be considered simultaneously. Experimentally rational design of pegRNA is complex, and the interactions between sequence elements are difficult to grasp empirically. To address these challenges, computational design methods adopt a data-driven strategy, enabling rational design through comparative analysis, efficacy prediction, and the extraction of underlying patterns, thereby empowering efficient and rapid pegRNA design.

Currently, three computational tools have been developed for rapid pegRNA design (Fig. 3C). Here, pegFinder enables efficient pegRNA design by comparing reference and edited DNA sequences, using a modified Needleman-Wunsch algorithm with affine gap penalties to identify sequence variations between the target and original sequences [84]. This approach evaluates both edit positions and sgRNA GC content to generate optimal editing sequences. Alternative platforms, PrimeDesign [86] and DeepPE [85], have been specifically optimized for designing edits relevant to human genetic diseases. Notably, DeepPE incorporates both traditional machine learning models and deep learning algorithms to achieve superior predictive performance. It is important to emphasize that pegRNA design remains in its nascent stages, where computational methods can provide valuable insights to guide rational design principles. However, further optimization and refinement of these tools will require continued investigation and iterative improvement to fully realize the potential of prime editing technology. The development of more sophisticated algorithms incorporating additional biological parameters and experimental validation will be essential for advancing this promising therapeutic approach (Fig. 3D). In addition, the interpretability of the design is gradually developing, enabling operation without reliance on predefined features [87].

Overall, computational design of CRISPR-associated RNAs is primarily directed toward controllable functional regulation through optimization of editing efficiency and specificity. Conventional gRNA design mainly involves sequence-level activity and off-target prediction, whereas pegRNA design additionally requires coordination of multiple functional sequence elements. Across both cases, model reliability depends strongly on the representativeness of experimental training and validation data. Moreover, these computational approaches mainly address guide-RNA design itself, while delivery of the RNA together with the required editing machinery constitutes a separate constraint on therapeutic implementation and is not explicitly captured by most sequence-design models.

## 3 Computational design for therapeutic mRNA: from sequence to function

mRNA therapeutics, as long-chain RNA molecules, function by facilitating intracellular expression of therapeutic proteins. mRNA therapeutics have emerged as a promising clinical modality due to their high precision and ability to enable *in situ* expression of therapeutic proteins without genomic integration [3]. These principles are particularly well illustrated in the design of mRNA vaccines, a major subclass of mRNA therapeutics. It is undeniable that mRNA vaccines are a promising platform in infectious disease prevention and cancer treatment [41].

**Fig. 4:**
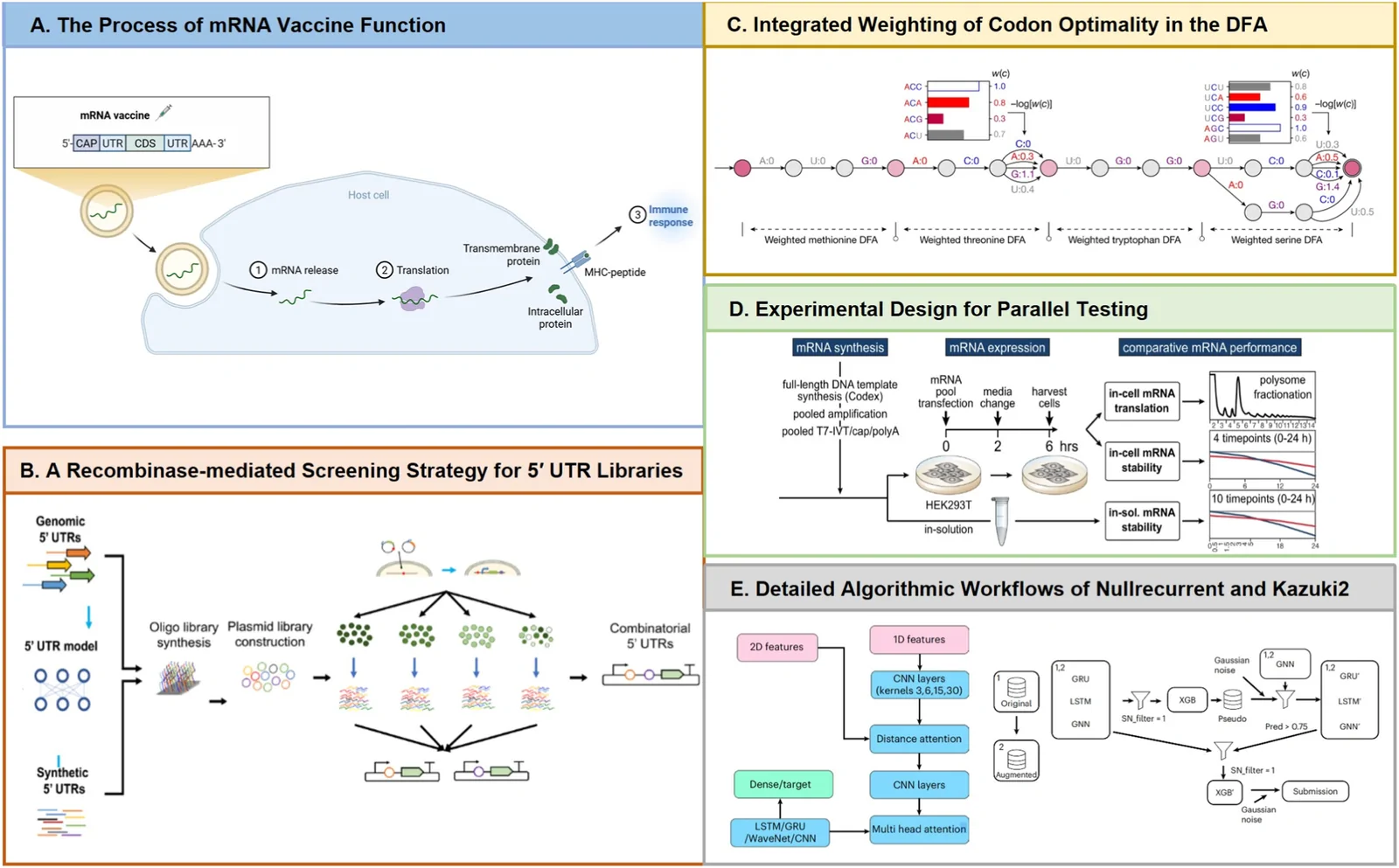
**Mechanisms of mRNA vaccines, sequence optimization strategies, and the construction and validation of computational models.** (A) The process of mRNA vaccine function. By expressing relevant immunogenic proteins in the cytoplasm, they activate T-cell immune responses to produce therapeutic effects. (B) A schematic overview of a recombinase-mediated screening strategy for 5′ UTR libraries. (C) Joint optimization of stability and codon optimality is achieved through integrated weighting of codon optimality in the DFA. (D) Experimental design for parallel testing of in-solution stability, intracellular stability, and ribosomal loading. (E) Detailed algorithmic workflows of two high-performing models, Nullrecurrent and Kazuki2. Panels adapted with permission from: B, ref. [88], under a Creative Commons license CC BY 4.0; C, ref. [25], under a Creative Commons license CC BY 4.0; D, ref. [23], under a Creative Commons license CC BY 4.0; E, ref. [24], under a Creative Commons license CC BY 4.0.

Unlike short RNA molecules, the multi-component and long-chain nature of mRNA renders its sequence design considerably more complex. The design of mRNA vaccines primarily focuses on optimizing several key structural components: the 5′ cap, untranslated regions (UTRs), open reading frame (ORF), and poly(A) tail (Fig. 4A). These elements collectively influence mRNA stability, translational efficiency, and immunogenicity. (1) the intrinsic instability and immunogenicity of mRNA molecules significantly limit their *in vivo* efficacy. (2) the translation efficiency of mRNA is highly dependent on its sequence and structural features. However, rational design rules for these characteristics remain underdeveloped, with distinct optimization principles required for each functional element. (3) localized mRNA optimizations may not necessarily lead to the global optimum in sequence or structure, necessitating a holistic approach to mRNA sequence design.

Currently, computational methods for designing specific mRNA sequences have proliferated, particularly for mRNA vaccine development. Given that long-chain RNA molecules can encode therapeutic proteins, optimizing various structural components to enhance immunogenicity presents a significant scientific challenge. By establishing algorithmic models, computational design can systematically decode the sequence-structure-function relationships of mRNA, enabling the rational optimization of its stability, translation efficiency, and immunomodulatory properties. This approach has substantially accelerated the development of mRNA-based therapeutics. Distinct computational design and optimization strategies have been developed for different mRNA elements (seeTable 3).

**Table 3:** **Computational methods used for mRNA design and prediction.** Specific topics, models, algorithms, datasets, input features, prediction endpoints, reported performance, validation evidence, code/web tool availability, and studies are organized.

Topic	Models	Algorithm	Datasets	Input Features	Prediction Endpoints	Reported Performance	Validation Evidence	Code/Web Tool Availability	Critical Assessment	Studies
5′ UTR design	Optimus 5-Prime	Convolutional neural network	Training database: 260,000 random 5′UTRs	One-hot encoded 1D 5′ UTR sequences	Measured mean ribosome load (MRL)	Optimus 5-Prime accounted for 93.4% of the variability in MRL	Held-out: 20,000 sequences withheld from the same eGFP reporter library; External: evaluation using 77 previously reported 5′ UTRs across six cell lines; Experimental/generalization: fluorescence validation of 10 selected UTRs and cross-reporter evaluation using an mCherry library	GitHub	Predicted and observed values eventual diverged at very high targeted ribosome loads	[22]
RNA degradation prediction and design	Nullrecurrent	Two sets of features into a single neural net architecture, combined elements of classic recurrent neural networks and convolutional neural networks	Training and testing database: ‘Roll-Your-Own-Structure’ Round I lab on Eterna by Leppek et al. [23]	Secondary structure nucleotide proximity	Three structural and autohydrolysis mapping profiles at single-nucleotide resolution	Private test set Mean Column RMSE of 0.34198, and Spearman correlation coefficient of 0.43 in predicting independent full-length mRNA degradation rates	Held-out/blind: OpenVaccine public and private test sets; External: independent PERSIST-seq dataset containing 188 full-length mRNAs	GitHub	Prediction accuracy heavily dependent on the performance of upstream structure tools	[24]
RNA degradation prediction and design	Kazuki2	Single-model neural networks with XGBoost	Training and testing database: ‘Roll-Your-Own-Structure’ Round I lab on Eterna by Leppek et al. [23]	Baseline 1D/2D RNA sequence and structure indicators	Quantitative prediction of SHAPE reactivity	Private test set Mean Column RMSE of 0.34266	Held-out/blind: OpenVaccine public and private test sets; External: independent PERSIST-seq dataset containing 188 full-length mRNAs.	GitHub	High structural complexity and parameter thickness due to hierarchical multi-level stacking	[24]
RNA design	LinearDesign	Deterministic finite-state automaton and beam search	Benchmark database: 114 human protein sequences from UniProt	Primary protein sequence and weighted finite-state automaton	A balanced mRNA sequence	57- to 128-fold increase in anti-spike IgG antibody titers in the reported experimental evaluation	Experimental: in-solution stability and cellular protein-expression assays; *in vivo* mouse immunogenicity evaluation	GitHub	Sacrificed some prediction accuracy to a certain extent	[25]
RNA design	DegScore	Ridge regression model	validation dataset: data from PERSIST-seq	single-nucleotide sequence identity windows	Predicting single-nucleotide resolution intrinsic autohydrolysis cleavage coefficients and general baseline decay constants in aqueous solution	Spearman R = −0.66, adjusted R = −0.67	External: independently generated PERSIST-seq mRNA measurements; Experimental corroboration: capillary-electrophoresis degradation measurements	GitHub	No weighted calculation was done for the modified sites	[23]

Within the three-barrier framework, mRNA represents a modality in which molecular stability, functional regulation, and delivery are particularly interdependent. At the molecular-design level, computational strategies mainly address three progressively integrated tasks: optimization of UTR-mediated stability and translation, optimization of ORF codon usage and structural properties, and whole-transcript design that balances multiple sequence and structural objectives. These tasks differ in their input features, computational formulations, and levels of experimental validation, while the large size and physicochemical properties of mRNA impose additional delivery constraints.

### 3.1 UTRs

Recognizing the impact of cell-specific RNA-binding proteins, the 5′ UTR and 3′ UTR elements of mRNA significantly influence its stability and intracellular translation [89]. Optimizing UTRs can therefore be crucial for maximizing mRNA translation yield in therapeutic and vaccine applications. The principal computational objective for UTR design is to identify sequence and structural features that regulate mRNA stability and translation in a context-dependent manner. However, optimizing UTRs faces three main challenges. (1) UTRs functional rules are highly complex and exhibit considerable cell-type specificity, making it difficult to design broadly applicable sequences. (2) the optimization space for UTRs is vast, particularly given the intricate interactions between RNA-binding proteins (RBPs) and cis-regulatory elements, which are not readily accessible through experimental methods alone. (3) UTRs’ function is influenced by secondary structure, and accurately predicting *in vivo* structural conformations remains a significant hurdle. To address these challenges, computational methods can integrate high-throughput data and employ machine learning models to predict complex relationships among sequences, structures, and functions. By deriving rational design principles from these mappings, it becomes possible to inversely engineer high-performance synthetic UTRs that surpass natural sequences, thereby accelerating the shift from experimental screening to rapid, rational design.

Recent studies have integrated high-throughput prediction and screening methods to optimize the 5′ UTR, enabling its rapid and systematic design. Cao et al. developed a high-throughput screening pipeline that first identifies naturally efficient 5′ UTRs, then applies genetic algorithms to predict optimized sequences (Fig. 4B) [88]. Using recombinase-mediated integration, they identified three synthetic 5′ UTRs that surpassed conventional non-viral gene therapy plasmids in efficiency. Additionally, Sebastian et al. introduced Optimus 5-Prime, a CNN model that predicts ribosome loading on the 5′ UTR to assess translation efficiency. The model demonstrated high accuracy in evaluating 20,000 test sequences. It should be noted that, while such models can predict 5′ UTR ribosomal loading with exceptionally high accuracy in large-sample random sequence tests *in vitro*, *in vivo* reproducibility is frequently compromised by data distribution shifts when applied to actual living tissues. They also developed Fast SeqProp (for straightforward sequence design) and Deep Exploration Networks (DEN) (for generating diverse, high-performance sequences), further advancing UTR optimization [22].

Targeting the 3′ end, the field is committed to developing high-performance synthetic UTR sequences that exceed their natural counterparts to meet the specific expression requirements of mRNA. In current clinical studies, researchers have been using the 3′ UTR of the human β-globin gene to achieve higher expression [90]. Nevertheless, ongoing research focuses on developing and exploring sequences that may outperform the existing UTRs. Orlandini von Niessen et al. have recently identified the AES-mtRNR1 3′ UTR, which exhibited significantly superior performance compared to human β-globin (up to 60-fold higher colony-forming units, compared to 6.5-fold). This was achieved through a multifunctional selection process known as SELEX (systematic evolution of ligands by exponential enrichment), conducted in a cell culture system, which facilitated a paradigm shift toward computational design and improved the success rate of wet-lab validation in preclinical non-viral gene therapy studies [91].

Advancements in massively parallel assays have deepened our understanding of how biological sequences influence molecular phenotypes, guiding rational design by elucidating stability-regulating mechanisms [92]. Leveraging this technology, Litterman et al. systematically analyzed over 25,000 RBP binding sites in mouse T cells. Their findings revealed that GC-rich 3′ UTRs tend to form secondary structures *in vivo*, reducing protein output by decreasing mRNA stability, half-life, and expression levels [93]. AU-rich elements are key cis-regulatory motifs in the 3′ UTR that modulate mRNA decay. Further research by Siegel et al. employed fast-UTR, a high-throughput functional annotation method, to analyze ARE-containing 3′ UTRs in human cell lines [94]. Their work uncovered novel features affecting ARE-mediated mRNA stability and expression, with implications for optimizing UTR design.

### 3.2 ORF

The ORF sequence determines the codon-encoded amino acid sequence of a protein. Due to the degeneracy of the genetic code, multiple codons can encode the same amino acid, but their translation efficiencies vary, leading to differences in protein yield and mRNA stability. This variation arises because codon selection influences ribosomal elongation rates—rare codons slow translation due to limited tRNA availability, while optimal codons enhance efficiency. Studies have shown that mRNA half-life correlates with codon optimality: introducing rare codons reduces stability, whereas optimizing codons significantly improves it [95]. Thus, deliberate codon optimization of the ORF is critical for maximizing mRNA performance. In contrast to UTR optimization, ORF design must preserve the encoded protein sequence while exploring synonymous sequence space to improve codon usage, translation efficiency, and mRNA stability.

To quantify codon bias, Sharp et al. developed the Codon Adaptation Index (CAI), which evaluates codon usage relative to highly expressed genes in a species [96]. However, CAI assumes a single “best” codon per amino acid, limiting its predictive accuracy. Addressing this, Nascimento et al. created the Gene Expression Codon Adaptation Index (geCAI), which directly links codon usage to transcript abundance using Spearman's rank correlation. In validation experiments, geCAI predicted GFP variant expression levels with 25-fold accuracy across varying mRNA concentrations, demonstrating its superiority for codon optimization [97].

Codon optimization has been pivotal in mRNA vaccine development. For example, Bhattacharya et al. [98] and Khan et al. [99] applied reverse vaccinology to design SARS-CoV-2 mRNA vaccines, using the Java Codon Adaptation Tool (JCat) for codon optimization [100]. JCat selects optimal codons while avoiding undesirable sequence features (e.g., restriction enzyme sites and transcription terminators), and calculates CAI values for uploaded gene sequences, streamlining ORF design. Such rule-based tools are particularly useful when codon adaptation must be performed under predefined sequence constraints, although they do not explicitly optimize the global secondary structure of the resulting mRNA. Coincidentally, a breakthrough by Baidu Research USA addressed the dual challenge of optimizing codon usage and mRNA secondary structure stability (Fig. 4C). By abstracting ORF codon lattice search as a classical computational linguistics problem and leveraging deterministic finite-state automata (DFA) in conjunction with dynamic programming algorithms, the approach jointly optimizes both codon optimality and secondary structural thermodynamic stability for the full-length mRNA antigen sequence of a COVID-19 vaccine within 11 min. Their algorithm, LinearDesign, simultaneously optimizes both parameters, identifying the most stable mRNA sequence for a COVID-19 vaccine in just 11 min. This data-driven algorithmic strategy not only substantially reduces the synthesis complexity of *in vitro* transcribed ORFs, but also overcomes the empirical and arbitrary nature of traditional design approaches through transcriptome-wide structural optimization. Consequently, it holds substantial translational significance for guiding the precise and rapid development of mRNA vaccines against emerging infectious diseases in preclinical settings [25].

Overall, computational ORF design has evolved from codon-frequency-based indices and rule-based optimization toward algorithms that jointly consider codon usage and RNA structural properties. The main inputs therefore extend from codon-frequency statistics to full coding sequences and secondary-structure-related objectives. Although experimental studies support the feasibility of such multi-objective optimization, the resulting designs remain dependent on the selected objective functions and biological context, and improvements in codon optimality or structural stability should not be interpreted as universally predictive of *in vivo* expression.

### 3.3 Overall sequence design and optimization

The comprehensive optimization of mRNA sequences necessitates a systematic approach that integrates multiple structural determinants of stability, translational efficiency, and functionality. This shift from element-specific optimization to whole-transcript design reflects a key limitation of independently optimizing UTRs or ORFs: improvements in individual components may not necessarily remain optimal when combined within the same full-length mRNA. While computational prediction methods offer valuable insights, their application is constrained by sequence length limitations, compounded by technical challenges in synthesizing full-length mRNA variants with diverse UTR-CDS combinations for experimental validation.

To address these limitations, Leppek et al. developed an innovative PERSIST-seq platform that enables parallel characterization of mRNA stability and translational efficiency through ribosome-loading analysis in human cells, both intracellular and in solution (Fig. 4D) [23]. Their findings establish intracellular mRNA stability as the predominant factor governing protein expression, with ribosome load contributing only modestly to overall output. Challenging the conventional overemphasis on translation initiation optimization, it points computational design toward new priorities.

Building upon these insights, Wayment-Steele et al. implemented an integrated design framework combining In-line-seq analysis with RiboTree optimization [101]. This approach strategically pairs translation-enhancing UTR elements (selected via DegScore) with structurally optimized coding sequences (designed using LinearDesign), reconciling the traditionally competing objectives of mRNA structural stability and protein yield. This integrated strategy is particularly relevant when UTR-mediated regulation and coding-region structure need to be optimized jointly rather than as independent design tasks. Its experimental validation supports the feasibility of balancing stability and translation-related objectives within the tested sequence and experimental context, although the resulting design relationships should not be assumed to generalize unchanged across different transcripts or cellular settings. By providing an experimentally validated framework, this work addresses the inherent challenges of balancing sequence stability, structural optimization, and translational output—a critical step toward the development of next-generation mRNA therapeutics. Furthermore, degradation prediction offers a viable approach for mRNA sequence design, demonstrating superior performance (Fig. 4E) [24]. These studies illustrate the increasing importance of experimentally grounded validation in whole-transcript design, where computationally optimized sequences are evaluated not only by model-derived scores but also by measured stability and translation-related phenotypes.

The 5′ cap and 3′ poly(A) tail are critical determinants of mRNA stability and translation efficiency. The 5′ cap serves as a protective structure at the 5′ end of the mRNA. It not only prevents mRNA degradation by exonucleases but also initiates translation and regulates pre-mRNA splicing and nuclear export [102]. The poly(A) tail significantly affects the mRNA's molecular lifetime. However, current strategies for mRNA element and sequence optimization remain predominantly focused on the UTRs and the ORF, which together account for over 90% of the total nucleotide sequence. Consequently, there is a pressing need to develop computational frameworks capable of the rapid simulation and design of diverse cap analogs and variable-length poly(A) tails. By exploring various cap analogs to elucidate their correlation with co-transcriptional efficiency, the translational potency of mRNA can be further enhanced [103]. Recent experimental advancements have yielded novel cap analogs based on cap-independent translation pathways, providing innovative solutions to enhance mRNA stability [104]. It is anticipated that the empirical data derived from these experimental iterations will establish a robust foundation for computational modeling, thereby accelerating progress in the field. Likewise, a mechanistic understanding of how poly(A) tail length modulates translation efficiency will provide the necessary theoretical support for computational methods to identify the “golden length” for optimal performance [105].

Nucleic acid foundation models and large language model (LLM)-inspired architectures are emerging as additional approaches for mRNA modeling. Unlike task-specific models trained primarily on labeled datasets, these approaches can use large-scale self-supervised pre-training to learn representations from diverse RNA sequences. Transformer-based RNA language models can learn sequence representations from large-scale pre-training and have been incorporated into downstream tasks such as RNA structural prediction [47]. However, their performance remains task- and benchmark-dependent, and their practical applicability is influenced by the scale and quality of pre-training data, task-specific fine-tuning, computational resources, and validation in the intended biological context. Accordingly, their value for mRNA design should be evaluated on a task-specific basis using appropriate downstream validation.

mRNA computational design illustrates a progression from element-specific optimization toward integrated, multi-objective transcript design. UTR models primarily address regulatory control of stability and translation, ORF optimization explores synonymous sequence space under protein-coding constraints, and whole-transcript approaches attempt to balance structural stability, translation-related properties, and interactions among multiple sequence elements. Across these levels, model performance depends strongly on the representativeness of training data and the type of experimental validation employed. Importantly, even an optimized mRNA sequence remains subject to delivery-dependent constraints, because transcript length, structure, modification state, and physicochemical properties can influence formulation and intracellular delivery.

## 4 Boundaries of computational design: other RNAs from sequence design to function applications

CircRNA and saRNA represent critical classes of other RNAs. As quintessential representatives of next-generation RNA therapeutics, circRNA and saRNA have emerged as research hotspots due to their advantages, including exceptional stability and sustained functionality. Compared with conventional mRNA, circRNA and saRNA introduce additional modality-specific design constraints arising from their distinct architectures and mechanisms of protein expression. For circRNA, computational design primarily involves balancing circularization efficiency, structural stability, and translation-related elements such as the Internal Ribosome Entry Site (IRES), whereas saRNA additionally requires coordination of replication-associated elements and prolonged expression. Thus, their design objectives, available input data, computational maturity, and validation evidence differ substantially. Beyond the existing hurdles in tertiary structure prediction, sequence optimization, and disease association modeling, additional complexities arise. These include the strategic selection of IRES sequences, the determination of optimal synthesis methodologies, and the precision engineering of replicase-encoding sequences. The complex structures render traditional trial-and-error experimental approaches both complicated and costly, underscoring the urgent need for computational design methods. To date, only a limited number of studies have addressed the development of computational methods for circRNA and saRNA.

### 4.1 CircRNA

**Fig. 5:**
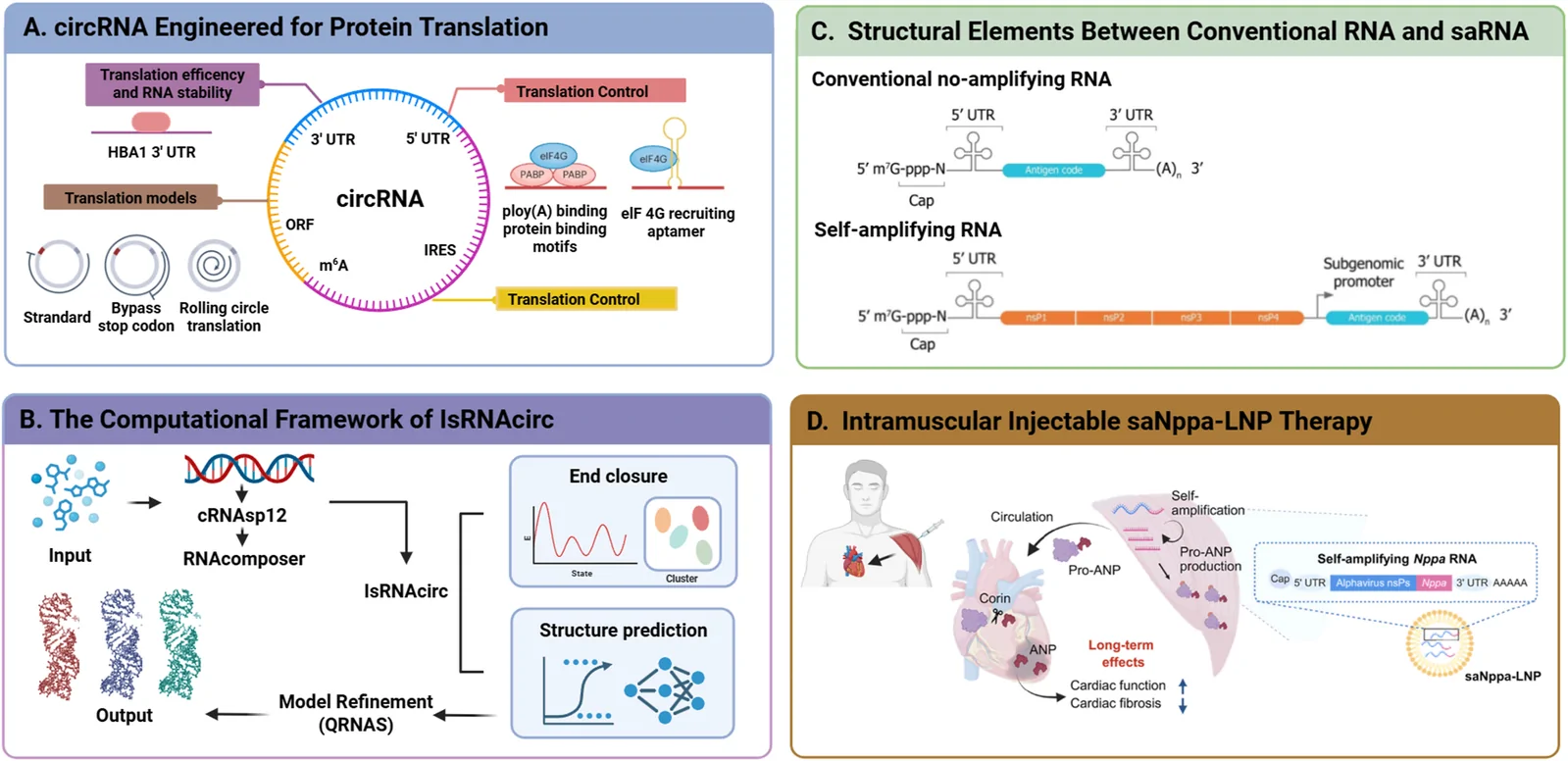
**Structural features, computational prediction, and therapeutic applications of circRNA and saRNA.** (A) circRNA engineered for protein translation. Key components include the 5′ UTR, 3′ UTR, IRES, *N*^*6*^-methyladenosine (m6A), and ORF. circRNAs mediate protein synthesis through distinct mechanisms, such as canonical ORF translation, stop codon bypass, and rolling circle translation. (B) The computational framework of IsRNAcirc enables three-dimensional structure prediction of circRNA. (C) Comparative analysis of structural elements between saRNA and conventional mRNA. SaRNA additionally encode four non-structural proteins (nsP1–4) and a subgenomic promoter to facilitate intracellular amplification. (D) Intramuscular injection of saNppa-LNPs promotes heart repair following myocardial infarction. Panels adapted with permission from: A, ref. [106], Springer Nature Limited; B, ref. [107], under a Creative Commons license CC BY 4.0; C, ref. [108], under a Creative Commons license CC BY 4.0; D, ref. [109], under a Creative Commons license CC BY 4.0.

CircRNA possess a unique covalent closed-loop structure, conferring a longer half-life compared to linear mRNAs (Fig. 5A). This endows circRNA with significant application potential in the therapeutic field [106]. However, the unique structure of circRNA relies on *in vivo* or *in vitro* circularization platforms, making the rapid and efficient production of circRNA a challenge. Furthermore, predicting circRNA three-dimensional structures and rapidly designing sequences still require computational methods. Consequently, computational methods for circRNA continue to be developed. The central computational objective for circRNA is not simply sequence optimization, but the simultaneous coordination of circularization efficiency, structural stability, and translation-related functionality.

CircRNA still follows the sequence-structure paradigm, and sequence design requires the coordination of three key indicators: efficient circularization, a compact structure that maintains stability, and preservation of IRES integrity. To balance these three factors, the circDesign algorithm was developed to design circRNAs with improved circularization efficiency and stability [110]. This method uses minimum free energy and CAI as indicators, and introduces IRES structural deviation as a constraint parameter. It quantifiably reduces interference between the IRES structure and other regions, preserving its native conformation to the greatest extent possible. This provides a generalizable strategy for circRNA design algorithms.

Determining circRNA structure is also a critical aspect of the design process. Structure prediction for circRNA has generally relied on extensions of linear RNA models. Given the limited availability of circRNA templates, *de novo* prediction methods can be employed. For instance, IsRNAcirc enables three-dimensional structure prediction of circRNAs through coarse-grained molecular dynamics simulations [107]. This model significantly reduces local irrational elements in the initial input, providing a feasible approach for capturing the complex structural features of circRNA (Fig. 5B). Additionally, secondary structure prediction for circRNA is gradually being developed. cRNAsp12, a representative tool in this area, employs a landscape partitioning approach to predict circRNA secondary structures within a limited folding landscape [111].

Beyond direct molecular design and structure prediction, computational analysis of circRNA also extends to functional interpretation, particularly the identification of disease-associated circRNAs. CircRNAs have been identified in a variety of organisms and tissues and are considered to be associated with the pathogenic mechanisms of various diseases. Therefore, predicting circRNA-disease associations has become a major area where computational methods play a significant role. To date, numerous models have been developed for circRNA-disease prediction, with approaches based on machine learning [112], deep learning [113], and graph embedding techniques [114] under certain objective functions emerging in rapid succession. Furthermore, multi-objective evolutionary algorithms have also improved accuracy in circRNA-disease prediction [115]. Overall, the prediction accuracy and the efficiency of design optimization still need further improvement [116].

Current circRNA computational studies span three related but distinct tasks: sequence-level design, structural prediction, and disease-association modeling. Their inputs therefore range from sequence and thermodynamic features to structural representations and heterogeneous biological association data, while the corresponding methods include constrained optimization, molecular simulation, machine learning, deep learning, and graph-based approaches. However, these tasks remain unevenly developed, and experimental validation of computationally optimized circRNA designs is still more limited than for conventional mRNA. In addition, most current models evaluate circRNA properties at the molecular or functional level without explicitly incorporating carrier compatibility or intracellular delivery, which remains a separate constraint on therapeutic application.

### 4.2 SaRNA

SaRNAs, derived from positive-sense RNA viruses, offer a compelling advantage over conventional mRNA therapeutics: their intrinsic replication capacity enables robust and sustained protein expression from minute doses (Fig. 5C) [108]. Unlike conventional mRNA, the design objective for saRNA extends beyond translation efficiency to the coordinated optimization of sequence features required for both RNA replication and downstream protein expression. Despite this potential, the computational design of saRNA remains largely uncharted territory. This gap is primarily attributable to the current scarcity of high-quality experimental datasets necessary for training predictive models. Moreover, the structural complexity of saRNA, which harbors multiple functional elements required for replication, introduces additional layers of difficulty for sequence optimization. Potential computational inputs therefore include not only conventional sequence and structural features but also replication-associated elements and their interactions. However, because sufficiently large and standardized saRNA datasets are currently lacking, these design variables have not yet been incorporated into broadly validated computational frameworks. The development of saRNA delivery systems [117] and their therapeutic applications is still ongoing, offering significant advantages for disease treatment (Fig. 5D) [109]. It is anticipated that more computational methods for saRNA will be developed and applied in the future.

Consequently, there is an urgent need for advanced computational frameworks to intervene in circRNA and saRNA engineering. By leveraging high-throughput screening integrated with computational algorithms, it becomes possible to achieve rapid optimization of individual modular components, ultimately yielding sequences that attain local or global optima in terms of both stability and translation efficiency.

## 5 Delivery system design and optimization

While molecular optimization can improve the intrinsic function of an RNA payload, therapeutic performance also depends on whether the resulting molecule can be effectively formulated, protected, delivered, and released into the appropriate intracellular compartment. RNA payload design and delivery-system design are therefore not entirely independent: payload properties can affect carrier assembly and delivery behavior, while formulation and intracellular-delivery constraints can in turn influence the choice of RNA architecture and molecular design. Before discussing carrier-centered computational optimization, we first consider the emerging evidence for payload-carrier interdependence and its implications for computational RNA therapeutic design.

### 5.1 Payload-carrier interdependence in RNA therapeutic design

Although RNA payload design and delivery-system optimization have historically been developed largely as separate tasks, accumulating evidence indicates that this separation is only an approximation. Payload properties, including RNA length, molecular structure, chemical modification, charge-related characteristics, and expression behavior, can influence formulation and delivery performance, while the carrier environment can in turn affect RNA structure, stability, and biological activity [[118], [119], [120], [121], [122], [123]]. At the computational level, however, systematic co-optimization of payload and carrier variables remains limited. One machine-learning framework has already incorporated payload-related descriptors, including self-amplification capability, cap structure, modification, and codon optimization, together with LNP formulation parameters within the same predictive model [26]. This example illustrates an emerging shift from independent optimization toward joint consideration of payload and carrier properties.

This interdependence is particularly evident at the physicochemical level. RNA length and the associated phosphate-backbone charge influence electrostatic complexation, payload loading, and the formulation space required for efficient encapsulation. Consistent with this, mRNA loading and distribution within LNP populations vary with lipid-to-RNA formulation conditions [118], and different mRNA lengths have been shown to favor different ionizable-lipid structures for efficient delivery [119]. These effects are further influenced by RNA structure: distinct RNA conformations exhibit different interactions with ionizable-lipid layers [120], while encapsulation itself can alter the structural state and stability of the RNA cargo in a lipid-composition-dependent manner [122]. Chemical modification introduces an additional layer of coupling, because the biological effect of nucleoside modification can vary with the chemistry of the LNP used for delivery [121]. Thus, sequence, structure, and chemical modification affect not only intrinsic RNA function but also the behavior of the complete RNA–carrier formulation.

Payload–carrier coupling also extends to RNA modality and expression kinetics. Direct comparisons of linear mRNA, circRNA, and saRNA delivered by lipid- and polymer-based carriers have shown that protein-expression profiles depend on both RNA architecture and carrier type [123]. This indicates that a carrier optimized for one RNA modality cannot necessarily be assumed to perform equivalently for another, particularly when payloads differ in length, structure, intracellular amplification, and intended expression duration. Conversely, delivery constraints can feed back into payload design, because changes introduced to improve molecular stability or expression may also alter encapsulation requirements, carrier compatibility, intracellular release, and final therapeutic output. Nevertheless, most current computational studies still treat the RNA payload as fixed or incorporate only a limited subset of payload descriptors, while focusing primarily on carrier-side optimization. Therefore, after establishing this payload–carrier interdependence, the following subsections review the more mature carrier-centered computational tasks, including formulation optimization, organ targeting, protein-corona and *in vivo* prediction, intracellular delivery, and alternative carrier design.

### 5.2 LNP formulation optimization

**Fig. 6:**
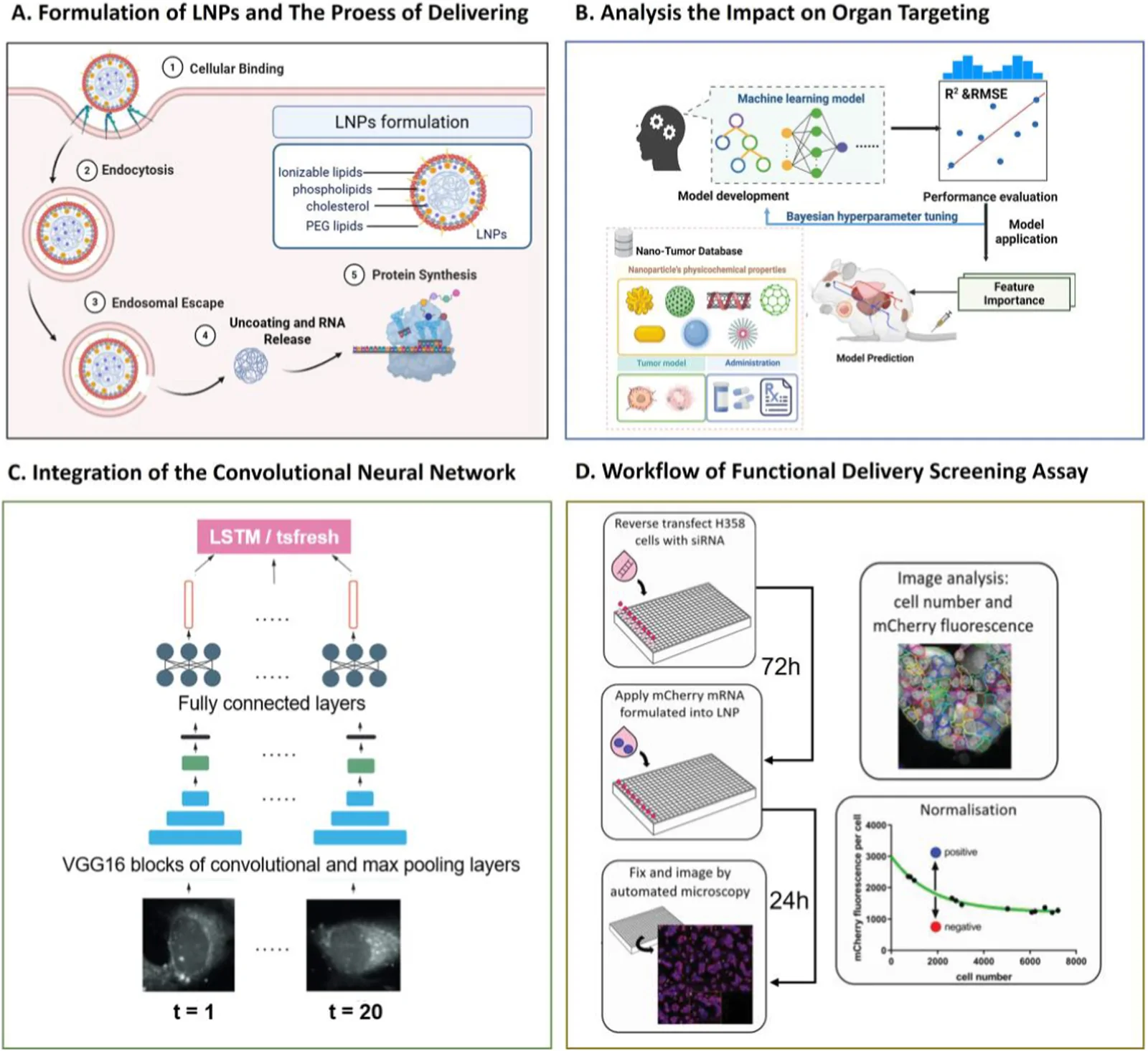
**Delivery mechanisms of LNPs, high-throughput screening, and machine learning-based optimization and prediction.** (A) Formulation of LNPs and the process of delivering RNA into cells for expression. Typically, LNPs consist of four components: ionizable lipid, phospholipids, cholesterol, and PEG-lipids. After entering the cell, LNPs must undergo endosomal escape to function in the cytoplasm. (B) A schematic illustration of the study framework for analyzing the impact on organ targeting using different delivery data. (C) Integration of the convolutional neural network to form a classification model. (D) Schematic workflow of functional delivery screening assay. Panels adapted with permission from: B, ref. [32] under a Creative Commons license CC BY 4.0; C, ref. [33], Informa UK Limited; D ref. [34] under a Creative Commons license CC BY 4.0.

Among available platforms, lipid nanoparticles (LNPs) have emerged as the most clinically advanced system, with their four-component architecture (ionizable lipids, phospholipids, cholesterol, and polyethylene glycol lipids) providing both protection and enhanced cellular delivery (Fig. 6A). The influence of LNP formulation and proportion on delivery effect requires extensive experimentation, which aligns with the computational model's prediction and optimization strategy. The multicomponent nature of LNPs presents significant formulation challenges, as the relative ratios of these components substantially impact RNA delivery efficiency and expression levels. However, empirical optimization through iterative synthesis and testing of different LNP compositions remains a tedious, time-consuming, and resource-intensive process. This limitation has driven recent efforts to develop computational methods to predict and optimize LNP performance parameters. By integrating computational methods such as machine learning with high-throughput data, predictive models can be established to accelerate the rational design of optimal LNP formulations and enable a mechanistic understanding of the key factors influencing the delivery process (seeTable 4).

**Table 4:** **Computational methods used for LNP design and prediction.** Specific topics, models, algorithms, datasets, input features, prediction endpoints, reported performance, validation evidence, code/web tool availability, and studies are organized.

Topic	Models	Algorithm	Datasets	Input Features	Prediction Endpoints	Reported Performance	Validation Evidence	Code/Web Tool Availability	Critical Assessment	Studies
IgG titer prediction	---	Hyper-parameters of lightGBM	325 formulation samples	Some different properties	Log-transformed binding IgG antibody titer	R^2^ > 0.90 for training and validation sets; 10-fold CV R^2^ > 0.87	Held-out: 65/325 same-source samples; Internal: 10-fold cross-validation; Experimental: mouse validation of selected formulations	From the authors	The small sample size leading to no further results	[26]
LNPs design	ANN-DOE	Six machine learning algorithms	18 experimental formulations generated using a definitive screening design (DSD)	3 continuous parameters and 2 continuous material attributes	4 critical quality attributes of mRNA-LNPs	ANN R^2^ = 0.7269-0.9946 across the evaluated CQAs	Internal/model evaluation within the study-specific DSD dataset	From the authors	Limited transferability beyond the evaluated formulation and process conditions	[27]
LNPs design	---	Random forest, logistic regression, and gradient boosting	*In vitro* mRNA transfection data for a total of 584 ionizable lipids	A compressed feature vector containing 2014 end-selected	Binary output mapping (“success” or “failure")	Exhibiting the highest receiver operating characteristic area under the curve (ROC-AUC) at 0.983	Prospective experimental validation: the top 16 ML-selected lipids were synthesized and experimentally tested	Online	Limitation in natively factoring post-translational exocytosis kinetics and systemic circulation exchange mechanics	[28]
Efficiency Prediction	---	XGBoost and shapley additive explanations (SHAP)	Published meta-analysis including 162 samples covering 23 tumor cell lines	6 nanoparticle properties, 3 tumor characteristics and 60–66 genes	Log-transformed delivery efficiency at 24/168 h and max postinjection	R = 0.66 (maximum), 0.75 (24 h), and 0.54 (168 h) on the test sets	Held-out test-set evaluation within the study dataset	GitHub	Heterogeneous nanoparticle–tumor dataset; limited specificity to RNA-LNP systems	[29]
Distribution Prediction	---	Random forest	652 literature-derived data points from 56 papers; 567 data points for functional-composition modeling	Initially spanning 21 total factors (8 qualitative and 13 quantitative descriptors)	Relative protein abundance of the protein corona	R^2^ > 0.75 for protein-corona prediction; most experimentally verified functional-composition models achieved R^2^ > 0.80	Internal: RF out-of-bag validation and 10-fold cross-validation; Experimental: laboratory analysis of Au- and Fe_3_O_4_-NP coronas and cellular recognition	GitHub	Exhibiting a strong class-imbalance dependency	[30]
Distribution Prediction	---	Supervised deep neural network	A total of 63,630 protein label-free quantitative intensity data points	Ion current intensities of 707 significant individual surface-adsorbed proteins	Nanoparticle *in vivo* transport and pharmacokinetic destiny outcomes	>94% accuracy for spleen/liver accumulation in a double-blinded study	Blinded experimental validation: double-blinded prediction of *in vivo* spleen and liver accumulation	Online	Easily influenced by datasets	[31]
Distribution Prediction	---	Deep neural networks	Nano-Tumor Database: 534 tumor datasets (2345 data points) and 1972 datasets for five major organs (8461 data points)	3 attribute categories,	Specific tissue delivery efficiency	R^2^ achieving 0.41 (tumor), 0.42 (heart), 0.45 (liver), 0.79 (spleen), 0.87 (lung), and 0.83 (kidney)	Internal: 5-fold cross-validation; Held-out: 20% test subset from the same Nano-Tumor Database	GitHub	Over-reliance on data	[32]
Cell expression prediction	---	Convolutional neural network, long short-term memory network and gradient boosting machines	Time-lapse data for 774 HepG2 cells	32-dimensional baseline single-cell latent feature vectors	Green fluorescent protein expression	Temporal models improved predictive performance compared with static-image features	Internal: 5-fold cross-validation on the training dataset; Held-out: 172-cell test set from the same imaging study	GitHub	Uncertain transferability across cell types and LNP formulations	[33]
LNPs design	ACE-ID	Random Forest, Gradient Boosted Tree, and K-Nearest Neighbor model	850 available phenotypic features	Multi-parametric phenotypic fingerprints	Functional mRNA delivery and translation efficiency	Overall F1 score = 0.74–0.81; class-specific accuracy = 0.66–0.75 using 10 selected phenotypic features	Internal/model evaluation within the phenotypic profiling dataset; Experimental: pathway perturbation and re-engineered MC3-LNP validation *in vitro* and *in vivo*	Online	Limited evidence for transferability across cell types and LNP formulations	[34]

The ratios of various components in LNPs exhibit complex, nonlinear relationships with their delivery efficiency, making it difficult to achieve global optimization solely through empirical approaches. Therefore, the impact of component ratios on mRNA delivery has been a major focus of recent research. Wang et al. developed a comprehensive machine learning model to predict optimal LNP formulations for antiviral mRNA vaccines [26]. Their model incorporated numerous input parameters, including antigen protein type, self-amplification capability, cap structure, pseudouridine modification status, codon optimization, and various lipid composition parameters (N/P ratio, ionizable lipid fraction, DSPC score, etc.). Using the Light Gradient Boosting Machine (lightGBM) algorithm, they achieved strong predictive performance (R^2^ > 0.87) for vaccine immunogenicity and established important relationships between ionizable lipid substructures and *in vivo* potency. Complementary work has shown that cationic ionizable lipids with N/P ratios ≥6 provide superior encapsulation efficiency [27]. Importantly, this study differs from carrier-only formulation models because several payload-level descriptors were incorporated together with LNP composition variables within the same predictive framework. It therefore provides an example of how computational modeling can begin to capture payload-carrier interactions, although the available descriptors still represent only a subset of the molecular and biological factors involved in RNA delivery. Furthermore, by integrating the physicochemical properties of LNPs with tumor genomic profiles, a machine learning-based model was developed to predict LNP delivery efficiency. Through interpretable analysis, this model elucidated the various factors influencing LNPs delivery to tumors, thereby advancing the development of personalized precision nanomedicine [29]. Computational models can extract key features from complex parameters to provide guidance for formulation optimization.

To further enhance the efficiency of formulation optimization, high-throughput synthesis (HTS) strategies can significantly accelerate the design and identification of novel ionizable lipids for RNA delivery (Fig. 6B) [28]. The research team developed an innovative HTS platform based on four-component reactions, which substantially improved the efficiency of ionizable lipid design and generation. To enhance both the quality and diversity of transfection data, they established a combinatorial library comprising 200 newly designed lipids, whose structures were determined through *in vivo* screening of LNP-mRNA formulations following intramuscular administration. Among various nonlinear models evaluated, the eXtreme Gradient Boosting (XGBoost) algorithm demonstrated superior performance and was consequently selected for screening a new library containing 40,000 untested lipid candidates. Using *in vitro* transfection results from 584 distinct LNPs as training data, the team developed and compared multiple machine learning models. Through this approach, they identified ionizable lipid 119-23, which exhibited significantly enhanced transfection efficiency across multiple tissue types (including muscle) and immune cells compared to established benchmark lipids.

### 5.3 Organ-targeting LNP design

Traditional LNPs are primarily focused on liver targeting, while achieving organ selectivity for extrahepatic targets remains an important challenge. Numerous experimental approaches have been developed to formulate LNPs for targeting different organs, mainly focusing on the optimization of ionizable lipids [124]. Among these, the SORT strategy is a representative method that adjusts the charge properties of lipids, such as permanently cationic lipids, anionic lipids, and ionizable cationic lipids, within the formulation to achieve organ-specific targeting [125]. Additionally, incorporating artificial ionizable and natural amino acids as functional components into peptide-ionizable lipids has been shown to enable controllable organ specificity [126]. Although these experimental approaches are feasible, the reliance on trial-and-error experiments significantly hinders the advancement of LNPs. Computational methods, with their unique capabilities in data analysis and effective data classification, have emerged as a key tool for predicting LNPs’ biodistribution and developing novel organ-specific LNP formulations.

### 5.4 Protein corona and *in vivo* prediction

Various machine learning strategies are being employed to guide the development of LNPs with organ-specific targeting capabilities, while also providing interpretable insights. Upon entering the bloodstream, LNPs interact with biological components to form a protein corona, which influences their physicochemical properties and organ distribution. Therefore, predicting the composition and function of the LNPs protein corona can facilitate the rational design of nanoparticle composition and structure. A random forest algorithm incorporating 10 independent and significant variables has demonstrated excellent performance in overcoming data heterogeneity and predicting protein corona composition [30]. Furthermore, supervised learning using protein corona data collected at different time points in circulation has been employed to predict LNPs' biodistribution and clearance patterns *in vivo* [31]. Studies further indicate that the dynamic changes and combinatorial patterns of the protein corona, rather than any single protein species, are critical determinants of LNPs’ behavior *in vivo*. These findings provide predictive models and theoretical support for modulating the organ-specific and circulatory lifetimes of LNPs. Deep neural networks have also emerged as a tool for predicting tumor and organ distribution. By mining and integrating existing literature data and using LNP formulation, size, and surface charge as variables, these models have successfully predicted nanoparticle distribution in tumors, heart, liver, spleen, lungs, and kidneys, offering an effective approach for developing LNPs targeting specific organs and tumors [32].

### 5.5 Cellular uptake and intracellular delivery prediction

A particularly challenging aspect of mRNA delivery is predicting successful cellular uptake prior to protein expression. At the cellular level, payload-carrier performance ultimately depends on successful uptake, intracellular trafficking, endosomal escape, and release of functional RNA into the cytoplasm. Current computational studies, however, predominantly model these processes from cellular or carrier-derived features rather than directly incorporating RNA sequence or structural descriptors. Harrison et al. addressed this challenge by employing time-lapse microscopy data combined with machine learning approaches (Fig. 6C) [33]. Their methodology involved using convolutional neural networks to extract cellular features at early time points, followed by analysis with long short-term memory networks or gradient boosting machines. Remarkably, their system achieved accurate prediction of GFP expression despite training on a relatively small dataset of only 774 cells, demonstrating the potential of computational approaches to overcome experimental limitations in delivery prediction. In a similar vein, Hunter et al. developed an advanced analytical platform termed ACE-ID (Advanced Cellular and Endocytic profiling for Intracellular Delivery) [34]. This approach utilized image analysis to obtain 850 phenotypic features from siRNA or compound-treated cell populations (Fig. 6D). Through machine learning analysis, they identified that only 10 key features were necessary for accurate predictions. The study employed multiple algorithms, including random forest, gradient boosting tree, and k-nearest neighbor models, to gain mechanistic insights into mRNA delivery by MC3-LNPs. These demonstrate the potential of computational models to utilize limited data in revealing the dynamics of the delivery process, thereby offering a novel approach for understanding and optimizing extracellular-to-intracellular transport.

### 5.6 Computational design of other delivery systems

Although lipid nanoparticles currently dominate the field of RNA delivery, advances in delivery systems are further exemplified by the rapid emergence of computationally driven novel carrier designs across multiple dimensions. Compared with LNPs, computational design of non-LNP RNA delivery systems is distributed across several mechanistically distinct carrier classes and remains uneven in maturity. Representative categories include polymeric carriers, ligand–oligonucleotide conjugates, and protein- or biologically derived carriers. These systems differ not only in material composition but also in their principal computational design variables and available data. Polymer-based models generally focus on molecular topology, charge density, polymer-RNA interaction, and complex stability; ligand-conjugate models emphasize receptor binding, uptake, intracellular trafficking, and pharmacokinetic-pharmacodynamic relationships; whereas protein-based carriers require consideration of macromolecular assembly, surface properties, cargo encapsulation, and tissue targeting. Consequently, model performance and validation cannot be compared directly across these carrier classes.

For polymeric carriers, computational design primarily addresses the large chemical and architectural search space of polymer-RNA complexes. Molecular descriptors such as polymer topology, molecular weight, charge density, and polymer–RNA interaction energetics can be combined with molecular dynamics or machine-learning approaches to prioritize candidate formulations. A representative case is the computational design of polymeric carriers, such as poly(β-amino ester) (PBAE). Leveraging computational virtual platforms, researchers have integrated coarse-grained molecular dynamics simulations with machine learning to compute the free energy landscapes of polymer-RNA complexes, enabling the combinatorial prediction of optimal polymer topologies and charge densities in silico, thereby overcoming the bottlenecks inherent in traditional trial-and-error experimentation [127].

Furthermore, computational design is continuously reshaping the composition of conventional lipid nanoparticles through the construction of novel carriers modified with polyhistidine. By systematically engineering molecular weight and linear or branched configurations, models can precisely predict protonation behavior within the acidic endosomal environment, thereby guiding the design of hybrid nanocarriers with potent endosomal escape capabilities [128].

Computational design has progressively transcended synthetic materials and advanced into the biological domain. Protein- and biologically derived carriers introduce another distinct computational space in which structural organization and biological function must be considered simultaneously. Through structural prediction and network-based design, researchers have successfully achieved the engineering of endogenous nanocage proteins. Computational models enable dynamic simulations of macromolecular protein assembly, surface charge distribution, and mRNA encapsulation capacity, thereby facilitating the design of naturally derived protein delivery systems with organ-targeting properties [129]. Compared with synthetic polymer libraries, however, the available datasets for these systems are generally smaller and more heterogeneous, and model development often depends on study-specific structural or experimental measurements. Although such approaches may provide mechanistic interpretability at the molecular level, independent validation across different RNA cargos, tissues, and biological environments remains limited.

Among non-LNP delivery strategies, ligand-oligonucleotide conjugates represent a therapeutically relevant category, with N-acetylgalactosamine (GalNAc)-conjugated siRNA for hepatocyte targeting as a primary example. Unlike nanoparticle-based encapsulation, this approach relies on asialoglycoprotein receptor (ASGPR)-mediated endocytosis, thereby linking ligand structure and oligonucleotide chemistry to receptor binding, intracellular trafficking, and pharmacodynamic outcomes. Nair and colleagues first demonstrated that multivalent GalNAc conjugation supports hepatocyte uptake and RNAi-mediated gene silencing after subcutaneous administration [130]. These experimentally established relationships between conjugate architecture, receptor-mediated uptake, and silencing efficacy provided a mechanistic basis for subsequent computational modeling of GalNAc-siRNA delivery.

Mechanistic minimal physiologically based pharmacokinetic-pharmacodynamic (mPBPK-PD) models based on ordinary differential equation (ODE) compartmental frameworks have been developed to describe systemic disposition, GalNAc-ASGPR binding and internalization, endosomal transport, RNA-induced silencing complex (RISC) loading, and downstream gene silencing, with parameter estimation and interspecies scaling enabling translation across preclinical species and humans [131]. Complementing these models, genome-wide CRISPR knockout screening identified RAB18 as an intracellular regulator of conjugated-siRNA activity, highlighting endosomal trafficking as an important variable for mechanistic delivery models [132]. This mechanistic framework was subsequently applied to givosiran, a GalNAc-conjugated siRNA, with the model incorporating saturable ASGPR-mediated uptake, receptor occupancy, intracellular persistence, and pharmacodynamic turnover [133]. Whole-body physiologically based pharmacokinetic-pharmacodynamic (PBPK-PD) models have further extended this framework to tissue distribution and sensitivity analysis, identifying endosomal release and intracellular degradation as influential determinants of *in vivo* activity [134]. However, these models remain dependent on parameter quality and compound-specific data, while uncertainties in ASGPR abundance, endosomal escape, extrahepatic distribution, and species-specific physiology continue to limit their generalizability and clinical predictive capacity.

Computational design of non-LNP delivery systems is currently characterized by strong carrier-class dependence rather than by a unified modeling framework. Polymeric carriers benefit from relatively tractable structure-property descriptors and virtual chemical libraries, but simulations may incompletely represent biological transport. Ligand-conjugate systems offer more mechanistically defined receptor-mediated pathways and can therefore support PK-PD or PBPK modeling, although their predictions depend strongly on compound-specific kinetic parameters and species-dependent physiology. Protein-based carriers provide rich structural information but generally suffer from smaller and less standardized datasets. Across these classes, validation also occurs at different levels, ranging from in silico molecular simulation and *in vitro* screening to animal pharmacology and, in selected conjugate systems, clinical pharmacokinetic-pharmacodynamic observations. These differences make direct comparison of numerical model performance inappropriate and highlight the need to evaluate each approach according to its carrier class, dataset, prediction endpoint, and level of experimental validation.

### 5.7 Translational limitations of computational delivery models

Despite increasing efforts to computationally predict RNA delivery performance, models developed using *in vitro* data or individual animal systems do not necessarily retain the same predictive reliability across species or in clinical settings. A major reason is that several dynamic *in vivo* determinants are only partially represented in current datasets and modeling frameworks. First, adsorption of serum proteins can dynamically remodel the biological identity of a delivery system, altering its surface properties, biodistribution, cellular interactions, and clearance relative to the assumptions made under simplified experimental conditions [135]. Second, species-dependent differences in nanoparticle uptake, mRNA translation, endocytosis, and inflammatory responses can limit the transferability of delivery outcomes from animal models to human systems [136]. Third, organ-level delivery is influenced by hemodynamics, vascular and tissue barriers, steric constraints, and other physiological factors that are frequently simplified in computational models, contributing to discrepancies between predicted organ targeting and observed *in vivo* pharmacokinetics [137]. These factors collectively illustrate that predictive performance obtained in a controlled dataset or experimental system should not be interpreted as equivalent to clinical predictability. Improving translational reliability will therefore require models to incorporate more physiologically relevant variables and to be evaluated through progressively more stringent validation across *in vitro*, animal, and, where available, human datasets.

## 6 Challenges and prospects

### 6.1 Challenges

#### 6.1.1 Limitations in data quantity and quality

**Fig. 7:**
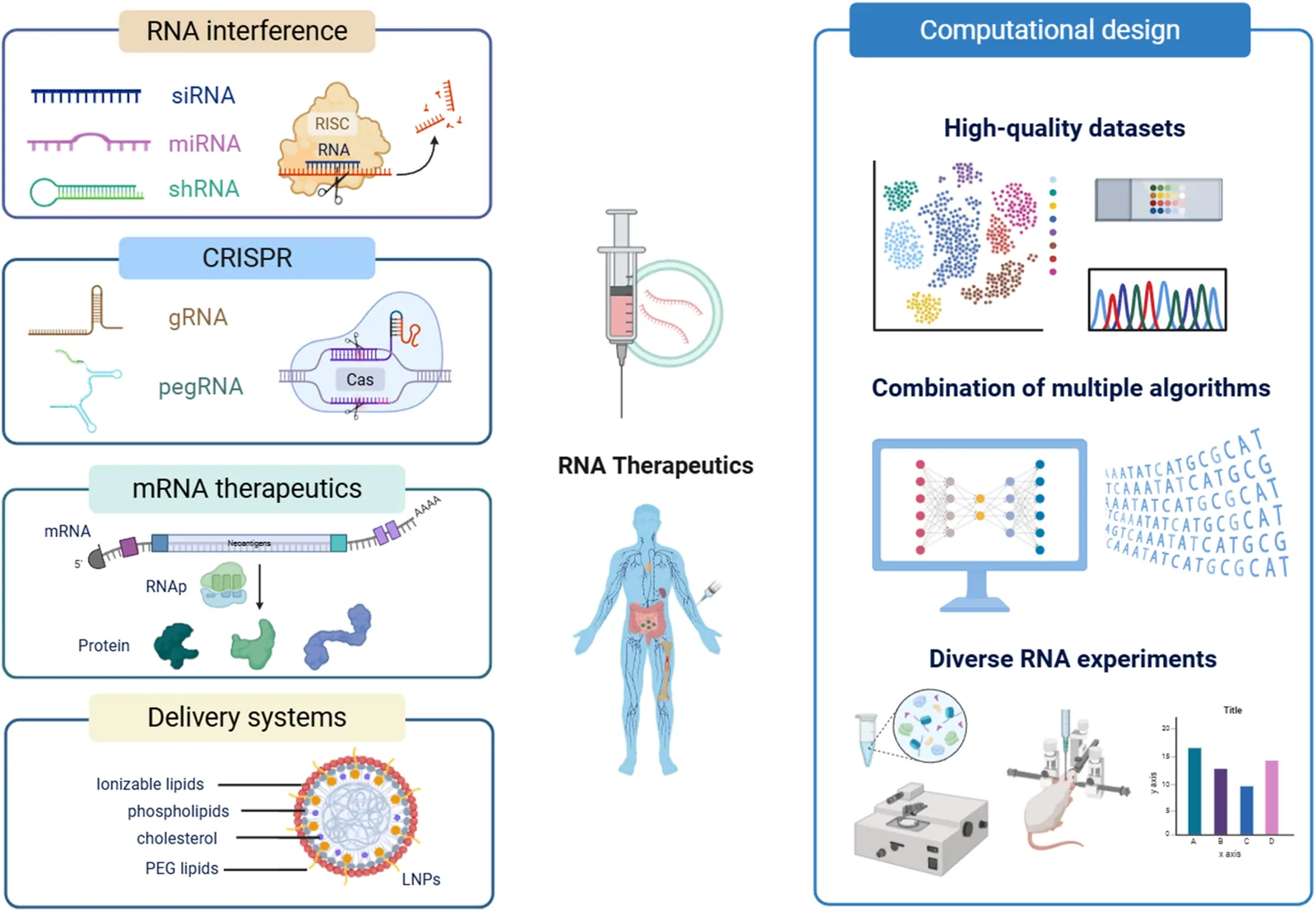
**Challenges and prospects in functional RNA.** Numerous machine learning algorithms have been developed for various types of RNA. The lack of high-quality datasets and insufficient adaptability of algorithms to different environments remain major obstacles restricting computational methods. With the development of various high-throughput experimental techniques for RNA, data on novel RNA species and chemically modified RNA are also increasing. Therefore, the refinement and high-quality processing and analysis of existing data represent crucial strategies for addressing these challenges. Computational methods will significantly advance and promote the application prospects of RNA therapeutics.

A variety of computational methods have been developed for the optimization of functional RNA design and the rapid screening of delivery systems. However, it must be emphasized that due to limitations in both data quantity and quality, there remains a significant disparity between computational predictions and practical applications, particularly in clinical drug development (Fig. 7). Substantial discrepancies exist between data derived from *in vitro* cell cultures and animal models compared to actual human physiological responses, making precise modeling extremely challenging. Current approaches are largely confined to processing and refining these limited datasets, presenting a particularly formidable obstacle for most algorithms - especially with the emergence and refinement of deep learning technologies. Consequently, the ability to extract sufficiently comprehensive and accurate information from constrained datasets has become a critical metric for evaluating and comparing algorithmic performance in this field.

#### 6.1.2 Insufficient adaptability in complex biological environments

The selection of optimal algorithms varies significantly across different experimental and clinical scenarios, representing an ongoing research challenge. While numerous computational approaches continue to emerge, the integration of multiple algorithmic advantages has become standard practice. Critical biological variables, including cell type specificity, animal model characteristics, RNA microenvironment, and modification patterns, differentially influence algorithmic performance. Consequently, traditional empirical algorithms often prove inadequate when confronted with environmental variations. Conversely, the development of robust, universally adaptable algorithms remains an urgent research priority. Three key metrics have emerged for evaluating algorithm efficacy: simplicity, precision, and interpretability.

#### 6.1.3 Lag in computational tools for diverse RNA structures and modifications

The unique structural and chemical properties of RNA present significant challenges for therapeutic development. RNA instability remains a major obstacle in RNA-based therapies, prompting exploration of structural modifications, including circRNAs, double-stranded RNAs (dsRNAs), and saRNAs. Notably, the design and optimization algorithms for these three RNA modalities remain largely underdeveloped. Incorporating modifications such as unnatural bases still lacks effective predictive and optimization methods, and many computational approaches do not account for these factors, leading to certain inaccuracies. The emergence of these novel RNA architectures has outpaced the adaptation of computational tools, creating a technological gap. Furthermore, accurate prediction of RNA stability and half-life remains an underdeveloped area. Current half-life determinations predominantly rely on indirect measurements through cellular translation assays, lacking precise analytical methods to directly assess RNA stability [138]. This data deficiency creates significant limitations for information-intensive algorithms, effectively rendering them “blind” to critical stability parameters.

### 6.2 Prospects

In the future, the development of computational methods for RNA sequence design, structural optimization, target prediction, delivery system design, and other aspects should encompass two key dimensions that collectively address the three critical challenges. On one hand, during the data generation process, it is essential to comprehensively capture specific RNA features, such as sequence information, modification sites, 5′ cap structure, 3′ poly(A) tail configuration, and delivery system composition in experimental settings. These features help refine various optimization parameters and enhance data quality, enabling accurate simulation and prediction of RNA in specific scenarios.

On the other hand, it is essential to focus on more effective utilization of existing data and enhancing the capacity for data analysis and processing. Data screening and optimization serve as crucial approaches for enhancing computational precision, while advanced characterization and analytical methods facilitate experimental validation of designed molecules. The emergence of spatial omics technologies and other novel techniques has significantly enriched RNA characterization approaches, partially mitigating discrepancies between computational predictions and actual biological contexts. At the algorithmic level, AI-based computation and prediction have injected new vitality into RNA therapeutics. Structural predictions and interaction analyses that were previously challenging can now be effectively addressed through AI methodologies. Notably, investigations into diverse RNA architectures, particularly circRNAs [139] and saRNAs [117], are emerging and demonstrating promising therapeutic potential. Therefore, the effective use of existing data can improve overall predictive performance for RNAs with diverse structures and functions. By integrating multiple algorithms to develop prediction models applicable across various scenarios, it is possible to improve data processing capabilities and address critical issues.

The continuous discovery of novel RNA structures and molecules imposes higher demands and broader prospects for computational design. For instance, chimeric RNAs, which contain exons from two different genes, are considered potential biomarkers for cancer diagnosis and therapy [140]. Their identification relies on bioinformatics mining to uncover possible disease associations. Furthermore, in studies of the mechanisms of formation and functional characterization of chimeric RNAs, effective knockdown of relevant genes is often required, necessitating rapid design of siRNA or shRNA using technologies such as RNAi. This presents extended applications for computational design. Additionally, mRNAs modified with both non-natural and natural bases exhibit enhanced translation efficiency within cells [141]. This necessitates that computational methods comprehensively consider factors such as modification types and sites during design to improve the expression of RNA molecules. The development of novel RNA molecules, such as L-RNA aptamers, also depends on information-processing techniques to enhance target specificity [142]. The ongoing evolution of RNA technologies will undoubtedly provide numerous promising avenues for the future of computational methods.

The development of intelligent technologies centered on RNA is propelling computational methods toward more cutting-edge and interdisciplinary directions. The delivery of RNA molecules is no longer confined to traditional lipid-based delivery systems like LNPs. A plethora of intelligent delivery technologies, represented by flexible electronic patches [143], which offer high efficiency and precise targeting, are continuously evolving. Computational methods must integrate with disciplines such as materials science and molecular design to provide model-based support for these corresponding intelligent delivery technologies, thereby accelerating their advancement. Furthermore, the inherent recognition potential of RNA is being explored in bioelectronics, where changes in electrical signals as the primary output modality still require support from computational models [144]. This illustrates the uniquely important role of computational methods in the development of RNA.

Based on the current methodological landscape, three future directions may be particularly relevant for computational RNA design.

(1) RNA foundation models and language-model-based architectures may increasingly complement task-specific machine-learning and deep-learning approaches by learning transferable representations from large sequence collections. Their potential, however, remains dependent on the downstream task, training-data composition, sequence and structural complexity, fine-tuning strategy, and validation setting. More broadly, current evidence does not establish that foundation models generally eliminate sequence-length limitations or reduce the in vitro-to-in vivo translation gap, and these questions remain important areas for further validation.
(2) Concerning design mechanisms, we envision a transition from static equilibrium thermodynamics assumptions toward dynamic kinetic simulations. Early computational tools tended to simplify RNA folding as spontaneous relaxation toward minimal free energy states under idealized conditions. However, long-chain RNA molecules in physiological environments frequently become trapped in kinetic folding traps or are constrained by steric hindrance. Through systematic synthesis and analysis of major computational approaches in sequence design, predictive modeling, and degradation profiling, we emphasize that future mechanistic frameworks should incorporate global-level kinetic simulations coupled with high-throughput analysis of weak bond dissociation probabilities across diverse topological configurations.
(3) Third, integrated RNA-carrier co-design represents a prospective direction in which payload and formulation variables are considered jointly rather than optimized in isolation. Existing studies already indicate that RNA length, structure, chemical modification, charge-related properties, and expression behavior can interact with carrier composition and delivery performance. However, systematic computational co-optimization of RNA payloads and delivery systems remains limited, and current models generally incorporate only a subset of these variables. Future co-design frameworks may therefore benefit from integrating molecular design, formulation properties, intracellular delivery, and pharmacological constraints within shared modeling strategies. Patient-specific RNA–delivery design should be regarded as a longer-term objective that will require computational prediction to be combined with experimental validation, manufacturing feasibility, pharmacology, and clinical evidence.

## 7 Summary

The advancement of computational tools has unlocked tremendous potential for RNA therapeutics. This review systematically examines computational methodologies for the design and optimization of three major classes of functional RNA, while also summarizing developments in delivery systems, particularly lipid nanoparticle platforms. The current paradigm for RNA design remains fundamentally rooted in the iterative triad of experimental data generation, rational design, and computational analysis. Notably, with the emergence of deep learning algorithms, predictive accuracy is no longer the sole evaluation metric - the capacity to adapt to diverse application scenarios has become an equally critical consideration in algorithm development.

The diversity of RNA types and structures poses significant challenges for prediction, design, and optimization. Therefore, the availability and accuracy of data become increasingly crucial, as data quality is the most critical determinant for computational methods. Moreover, the integration of design algorithms applicable to diverse RNA categories remains under development. Nevertheless, it is foreseeable that large language models will gradually assume a prominent role in RNA design and optimization, leveraging their inherent algorithmic mechanisms and their ability to rapidly meet specific user objectives [145].

Overall, as the life sciences advance, technologies targeting the RNA level will continue to develop. RNA therapeutics for various diseases are rapidly emerging. Computational methods are expected to remain important components of RNA therapeutic design as experimentally validated datasets and modeling approaches continue to expand. Continued integration of computational prediction with experimental and translational validation will be important for advancing RNA therapeutic development.

## CRediT authorship contribution statement

**Yiming Wang:** Writing – original draft, Visualization, Validation, Methodology, Investigation, Formal analysis. **Shengxin Tong:** Methodology, Investigation. **Xin Wang:** Methodology, Investigation. **Xiaowen Jin:** Methodology, Investigation. **Duoduo Tan:** Methodology, Investigation. **Yuan Lu:** Writing – review & editing, Supervision, Investigation, Funding acquisition, Conceptualization.

## Data availability

Data will be made available on request.

## Ethics approval and consent to participate

This manuscript is a literature review work, and thus no *in vivo* evaluations on animal model or clinical trials were performed in this scope. Thereby, our work does not fall into the incidence of ethical approvals and patient consents.

## Declaration of competing interest

The authors declare that they have no known competing financial interests or personal relationships that could have appeared to influence the work reported in this paper.
